# The physical and qualitative analysis of fluctuations in air and vapour concentrations in a porous medium

**DOI:** 10.1098/rsos.171954

**Published:** 2018-05-16

**Authors:** Y. Poorun, M. Z. Dauhoo, M. Bessafi, M. K. Elahee, A. Gopaul, A. Khoodaruth

**Affiliations:** 1 Department of Mathematics, University of Mauritius, Reduit, Mauritius; 2 Department of Mechanical and Production Engineering, University of Mauritius, Reduit, Mauritius; 3 Laboratoire d’Energétique, d’Electronique et Procédés, Université de la Réunion, , La Réunion

**Keywords:** fluctuations, steady state, linearization, relaxation–transport–diffusion, air-vapour concentration, qualitative and numerical analysis

## Abstract

This work presents the development and physical analysis of a sweat transport model that couples the fluctuations in air and vapour concentrations, and temperature, in a one-dimensional porous clothing assembly. The clothing is exposed to inherent time-varying conditions due to variations in the body temperature and ambient conditions. These fluctuations are governed by a coupled system of nonlinear relaxation–transport–diffusion PDEs of Petrovskii parabolic type. A condition for the well-posedness of the resulting system of equations is derived. It is shown that the energy of the diffusion part of the system is exponentially decreasing. The boundedness and stability of the system of equations is thus confirmed. The variational formulation of the system is derived, and the existence and uniqueness of a weak solution is demonstrated analytically. This system is shown to conserve positivity. The difficulty of obtaining an analytical solution due to the complexity of the problem, urges for a numerical approach. A comparison of three cases is made using the Crank–Nicolson finite difference method (FDM). Numerical experiments show the existence of singular coefficient matrices at the site of phase change. Furthermore, the steady-state profiles of temperature, air and vapour concentrations influence the attenuation of fluctuations. Numerical results verify the analytical findings of this work.

## 1. Introduction

Porous media has been a vital subject of research in recent years with applications spanning from scientific to engineering fields. One such area of interest is the movement of sweat in the form of vapour and air through textile fabrics. The behaviour of clothing systems under various environmental conditions is of utter importance. Clothing does not solely cover the human body, but also protects and provides thermal comfort to the body. It is necessary to choose the appropriate textile to suit the external environment. Various structural properties including the choice between hygroscopic and non-hygroscopic fabric plays a major role in the dissemination of sweat through the clothing system. Studies of the transport structure in fibrous porous textiles have been published from the 1930s. Over the years, many numerical models have been presented with improved modelling and more realistic features.

Henry [1,2] studied the diffusion of moisture in cotton bales for the first time. A linear coupled system of equations with respect to vapour and temperature was formulated. To keep the equations simple, some unrealistic assumptions were made. The diffusion constant was kept independent of the vapour concentration and temperature. It was not until the 1980s that significant progress was made in this field in terms of theoretical modelling and numerical analysis. The analysis of the convection–diffusion mechanism together with phase change in a porous slab was conducted by Ogniewicz & Tien [3]. Limited to a one-dimensional steady-state formulation, the humidity conditions were varied on both sides of the slab. A quasi-steady state model introduced by Motakef & El-Masri [4] was later extended by Shapiro & Motakef [5] to incorporate time dependence on heat and moisture transport. However, the application of this model was restricted to the choice of a larger advection time scale compared to that of diffusion. Further development was noticed in the works of Farnworth [6], Vafai & Tien [7] and Fan *et al*. [8].

A sweating guarded hot plate was used to conduct experiments on the distribution of temperature and water content in a three-layer clothing assembly comprising an inner fibrous batting sandwiched between an inner and outer thin covering fabric [9]. The experimental values obtained with a polyester batting have been used in this paper. This paper focuses on the work Huang *et al*. [10]. A more realistic model was presented where vapour and air motion are treated as separate components. An analysis of the time scale was conducted to understand the occurrence of convection, conduction, phase change and diffusion. Owing to a much shorter time scale compared to that observed during experiments, a quasi-steady system of equations for the vapour and air concentrations were chosen.

However, in reality air and vapour do not have such a smooth profile as shown in the numerical experiments of Huang *et al*. [10]. There always exists some fluctuation in the body temperature and external environment which, in turn, influences the concentration of vapour and air in the clothing. The main aim of this paper is to add an unsteadiness in terms of fluctuations to the air and vapour concentrations in the batting. Such a study provides an insight on the mechanism of relaxation, advection and diffusion with respect to air and vapour concentrations. The variables at steady state can be linearized to simplify the existing system of equations. A qualitative analysis is made on the nature of the solution to this problem. Theoretical analysis on the system of equations for the transfer of heat and moisture in porous fabrics can be found in Wang & Sun [11] and Kelly [12]. To the best of our knowledge, to date the system has not been treated with fluctuations. In this work, a qualitative analysis of the resulting coupled nonlinear equations with fluctuations is carried out. Each of the processes of relaxation, transport and diffusion, respectively, is influenced by fluctuations in both water vapour concentration (${\hat{c}}_{1}$) and air concentration (${\hat{c}}_{2}$). It is observed that the cross effect of the fluctuating variables on each other plays a significant role in the model. The coefficients of the matrices in the resulting relaxation–advection–diffusion equation have been found to influence the solution qualitatively, but at the same time add to the difficulty of the qualitative analysis. The existence of a positive and unique solution eliminates the need of a numerical approach to validate the new system of equations. However, the numerics are considered owing to the difficulty of finding an analytic solution. Varying situations at the inner and external environments are taken into consideration to understand the importance of the linearized profile taken for vapour and air concentration at steady state. The behaviour of the system of equations under different environmental conditions is tested using a semi-implicit finite difference scheme.

## 2. Mathematical description

**Figure 1.**
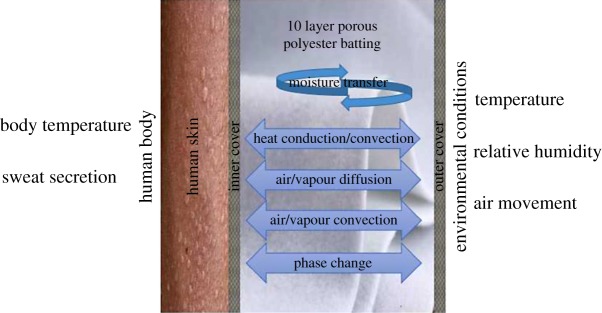
Schematic diagram of the clothing assembly.

Figure 1 shows the clothing assembly used in the experiments of Fan *et al*. [9]. Here, the batting of this assembly is studied under different internal and external conditions. Heat moves by convection in gas and by conduction in all phases. The vapour/air mixture is transferred through the porous textile media by convection and diffusion. Finally, phase change is induced by absorption of the fibre, evaporation and condensation. The direction of the above-mentioned processes depends on the internal and external environments.

These processes are governed by the equations (2.1)–(2.4), which model heat and mass transfer through porous media. They are acquired from the balance of mass, balance of energy and Fick’s law. These equations proposed in Huang *et al*. [10] are employed as an initial scenario in this work.

2.1 $$\begin{array}{r} \epsilon \frac{\partial C_{v}}{\partial t}+\epsilon \frac{\partial }{\partial x}(u_{g}C_{v})=\frac{D_{g}\epsilon }{\tau_{c}}\frac{\partial }{\partial x}\left(C\frac{\partial }{\partial x}\left(\frac{C_{v}}{C}\right)\right)-\Gamma , \end{array}$$

2.2 $$\begin{array}{r} \epsilon \frac{\partial C_{a}}{\partial t}+\epsilon \frac{\partial }{\partial x}(u_{g}C_{a})=\frac{D_{g}\epsilon }{\tau_{c}}\frac{\partial }{\partial x}\left(C\frac{\partial }{\partial x}\left(\frac{C_{a}}{C}\right)\right), \end{array}$$

2.3 $$\begin{array}{r} C_{vt}\frac{\partial T}{\partial t}+\epsilon C_{vg}\frac{\partial }{\partial x}(u_{g}T)=\kappa \frac{\partial }{\partial x}\left(\frac{\partial T}{\partial x}\right)+\lambda_{T}M\Gamma \end{array}$$

2.4 $$\begin{array}{r} \;\text{and}\;\rho (1-\epsilon^{\prime })\frac{\partial \tilde{W}}{\partial t}+\rho_{w}\frac{\partial u_{w}}{\partial x}=\rho (1-\epsilon^{\prime })D_{l}\frac{\partial }{\partial x}\left(\frac{\partial \tilde{W}}{\partial x}\right)+M\Gamma_{ce}, \end{array}$$

where *C*, *C*_v_ and *C*_a_ are the total, vapour and air concentrations (mol m^−3^), respectively, *T* is the temperature (K) and $\tilde{W}$ is the percentage liquid content on the fibre surface. *D*_g_ and *D*_l_ are the gas and liquid diffusion coefficients (m^2^ s^−1^), and *u*_*g*_ and *u*_*w*_ represent the vapour–air mixture and water velocity (m s^−1^) given by Darcy’s Law, respectively. *C*_*vt*_ and *C*_*vg*_ are the effective volumetric heat capacity of the batting and volumetric heat capacity of the gas mixture (J (m^−3^ K^−1^)). $\Gamma =\Gamma_{s}+\Gamma_{ce}\;(\mathrm{kg}\;(s^{-1}\;m^{-3}))$ is the phase change due to absorption, condensation and evaporation, and λ_*T*_ (J kg^−1^) is the latent heat of phase change. *ρ* and *ρ*_w_ are the densities for fibre and water (kg m^−3^). *κ* (W (m^−1^ K^−1^)) is the effective thermal conductivity, *τ*_c_ is the effective tortuosity of the batting and *M* (kg mol^−1^) is the molecular weight of water. The relationship between the porosity with water content, *ϵ*, and without water content, *ϵ*′, is given by $\epsilon =\epsilon^{\prime }-(\rho /\rho_{w})\tilde{W}(1-\epsilon^{\prime })$.

A few assumptions are made based on the nature of the problem under investigation and these are described in the next subsection.

### 2.1. Assumptions

As the textile fabric being used in the batting is polyester, which is non-hygroscopic, the liquid absorption by the fibre can be neglected. The resulting non-dimensional equations are as follows:

2.5 $$\begin{array}{r} {\left(c_{1}\right)}_{\tau }-v_{G}{\left(c_{1}{\left(c\theta \right)}_{\xi }\right)}_{\xi }=\delta_{G}{\left(c{\left(\frac{c_{1}}{c}\right)}_{\xi }\right)}_{\xi }-\frac{\beta_{G}}{\epsilon }S, \end{array}$$

2.6 $$\begin{array}{r} {\left(c_{2}\right)}_{\tau }-v_{G}{\left(c_{2}{\left(c\theta \right)}_{\xi }\right)}_{\xi }=\delta_{G}{\left(c{\left(\frac{c_{2}}{c}\right)}_{\xi }\right)}_{\xi } \end{array}$$

2.7 $$\begin{array}{r} \;\text{and}\;{\left(\theta \right)}_{\tau }-\frac{v_{T}\epsilon }{c_{vt}}{\left(\theta {\left(c\theta \right)}_{\xi }\right)}_{\xi }=\frac{\delta_{T}\kappa_{T}}{c_{vt}}\theta_{\xi \xi }+\frac{\beta_{T}}{c_{vt}}S, \end{array}$$

where *θ*, *c*, *c*_1_ and *c*_2_ are the non-dimensionalized terms for temperature, total concentration, vapour and air concentration, respectively, for 0≤*ξ*≤1. *ξ* and *τ* are, respectively, the non-dimensionalized spatial and temporal components. In addition, *c*=*c*_1_+*ϕc*_2_, where the constant *ϕ* comes from the scalings used to non-dimensionalize *C*_v_ and *C*_a_. The non-dimensionalization technique has been detailed in Huang *et al*. [10], and the values of the parameters can be found in table 1.

**Table 1.** Non-dimensional values of parameters for a 10-layer polyester batting.

parameter	value	parameter	value
*v*_*G*_	1.58×10^1^	*v*_*T*_	1.40×10^−2^
*δ*_*G*_	8.60×10^−3^	*δ*_*T*_	3.18×10^−5^
*β*_*G*_	3.30×10^1^	*β*_*T*_	6.4×10^−3^(*T*≥273)
			7.3×10^−3^(*T*<273)
*ϵ*=*ϵ*′	0.993	*ρ*_*fw*_	1.39
*α*	1.4258×10^1^	*p*_*s*0_	0.228
*θ*_*c*_	1.009	*c*_*vt*_	1.0261×10^4^
*κ*_*T*_	0.2553	*ϕ*	2.889952067×10^1^

Note that

2.8 $$S=A\frac{c_{1}\theta -p_{s}(\theta )}{\sqrt{\theta }},$$

with

2.9 $$A=(1-\epsilon^{\prime })$$

and

2.10 $$p_{s}(\theta )=\left\{ \begin{array}{ll} 0.675p_{s0}(\exp(1.37\alpha (\theta -1))-0.0538) & \mathrm{for}\;\theta <\theta_{c},\\ p_{s0}(\exp(\alpha (\theta -1))-0.412) & \mathrm{for}\;\theta \geq \theta_{c}. \end{array}\right.$$

Looking at the advection coefficient of temperature *v*_*T*_/*c*_*vt*_ which is approximately of order 10^−6^ whereas that of both concentrations (vapour and air) *v*_*G*_=15.8, the dynamical evolution of *θ* is completely different from that of *c*_1_ and *c*_2_. The time scale for the evolution of air and vapour concentration is not comparable to the temperature time scale. Thus, it can be deduced that *θ* does not vary about the steady state. The fluctuations in temperature are very small compared to those of air and vapour concentration. As a result, a stationary temperature is maintained within the polyester batting.

### 2.2. Fluctuations

The human body and environment are subject to changes in temperature, air movement and humidity among others throughout the day. Even in a healthy person, the body temperature oscillates according to the time of the day and depending on their exposure [13]. The core temperature fluctuates by approximately 0.6^°^C and is lowest at approximately 03.00, and highest at approximately 06.00 [14]. These fluctuations in temperature, in turn, cause fluctuations in air and vapour concentrations.

To take into account the fluctuations in *c*_1_ and *c*_2_, *c*_1_(*ξ*,*τ*) and *c*_2_(*ξ*,*τ*) are rewritten as

2.11 $$c_{1}(\xi ,\tau )={\bar{c}}_{1}(\xi )+{\hat{c}}_{1}(\xi ,\tau )$$

and

2.12 $$c_{2}(\xi ,\tau )={\bar{c}}_{2}(\xi )+{\hat{c}}_{2}(\xi ,\tau ),$$

respectively. ${\bar{c}}_{1}$ and ${\bar{c}}_{2}$ are the solutions at the steady state, and ${\hat{c}}_{1}$ and ${\hat{c}}_{2}$ are the fluctuations. It is assumed that ${\hat{c}}_{1}\ll {\bar{c}}_{1}$ and ${\hat{c}}_{2}\ll {\bar{c}}_{2}$.

Different situations are analysed with the aim of realizing the importance of environmental factors in the evolution of air and vapour concentration in the 10-layer polyester batting.

## 3. Methodology

Incorporating (2.11) and (2.12) in (2.5) and (2.6) gives the type of phenomena to be studied. The resulting system of equations is a nonlinear relaxation–transport–diffusion partial differential equation. The name Petrovskii parabolic has been given to such types of PDEs [15]. This equation governs the processes involved in the evolution of air and vapour in the porous batting. To study and understand the relaxation, transport and diffusion phenomena, the resulting equation is linearized about the steady-state values. The interactions between the different phenomena and between air and vapour are thus studied in equation (3.1). The presence of the total concentration at steady state, $\bar{c}$, in most of the coefficient terms of (3.1) indicates a strong coupling between the evolution of the fluctuations in concentration of air and vapour in the batting. Equation (3.1) indicates that the steady state plays a vital role in the evolution of ${\hat{c}}_{1}$ and ${\hat{c}}_{2}$.

3.1 $${\left(\begin{array}{c} {\hat{c}}_{1}\\ {\hat{c}}_{2} \end{array}\right)}_{\tau }=r\;\left(\begin{array}{c} {\hat{c}}_{1}\\ {\hat{c}}_{2} \end{array}\right)+t\;{\left(\begin{array}{c} {\hat{c}}_{1}\\ {\hat{c}}_{2} \end{array}\right)}_{\xi }+d\;{\left(\begin{array}{c} {\hat{c}}_{1}\\ {\hat{c}}_{2} \end{array}\right)}_{\xi \xi },$$

where

$$\begin{array}{rl} r & =\left(\begin{array}{cc} \begin{array}{l} v_{G}(\bar{c}{{}_{1}}_{,\xi }{\bar{\theta }}_{\xi }+\bar{c}{\bar{\theta }}_{\xi \xi }+{\bar{c}}_{1}{\bar{\theta }}_{\xi \xi }+{\bar{c}}_{\xi \xi }\bar{\theta }\\ \;+\;2{\bar{c}}_{\xi }{\bar{\theta }}_{\xi })+\delta_{G}(\frac{{\bar{c}}_{1}{\bar{c}}_{\xi \xi }}{{\bar{c}}^{2}}+\frac{\bar{c}{{}_{1}}_{,\xi }{\bar{c}}_{\xi }}{{\bar{c}}^{2}}\\ \;-\frac{2{\bar{c}}_{1}{\bar{c}}_{\xi }^{2}}{{\bar{c}}^{3}}-\frac{{\bar{c}}_{\xi \xi }}{\bar{c}}+\frac{{\bar{c}}_{\xi }^{2}}{{\bar{c}}^{2}})-\frac{\beta_{G}}{\epsilon }A\sqrt{\bar{\theta }} \end{array} & \begin{array}{l} v_{G}\phi (\bar{c}{{}_{1}}_{,\xi }{\bar{\theta }}_{\xi }+\bar{c_{1}}{\bar{\theta }}_{\xi \xi })\\ \;+\;\delta_{G}\phi \left(\frac{{\bar{c}}_{1}{\bar{c}}_{\xi \xi }}{{\bar{c}}^{2}}+\frac{\bar{c}{{}_{1}}_{,\xi }{\bar{c}}_{\xi }}{{\bar{c}}^{2}}-\frac{2{\bar{c}}_{1}{\bar{c}}_{\xi }^{2}}{{\bar{c}}^{3}}\right) \end{array}\\ \begin{array}{l} v_{G}(\bar{c}{{}_{2}}_{,\xi }{\bar{\theta }}_{\xi }+\bar{c_{2}}{\bar{\theta }}_{\xi \xi })\\ \;+\;\delta_{G}\left(\frac{{\bar{c}}_{2}{\bar{c}}_{\xi \xi }}{{\bar{c}}^{2}}+\frac{\bar{c}{{}_{2}}_{,\xi }{\bar{c}}_{\xi }}{{\bar{c}}^{2}}-\frac{2{\bar{c}}_{2}{\bar{c}}_{\xi }^{2}}{{\bar{c}}^{3}}\right) \end{array} & \begin{array}{l} v_{G}(\phi \bar{c}{{}_{2}}_{,\xi }{\bar{\theta }}_{\xi }+\bar{c}{\bar{\theta }}_{\xi \xi }+\phi {\bar{c}}_{2}{\bar{\theta }}_{\xi \xi }+{\bar{c}}_{\xi \xi }\bar{\theta }\\ \;+\;2{\bar{c}}_{\xi }{\bar{\theta }}_{\xi })+\delta_{G}(\phi \frac{{\bar{c}}_{2}{\bar{c}}_{\xi \xi }}{{\bar{c}}^{2}}+\phi \frac{\bar{c}{{}_{2}}_{,\xi }{\bar{c}}_{\xi }}{{\bar{c}}^{2}}\\ \;-\frac{2\phi {\bar{c}}_{2}{\bar{c}}_{\xi }^{2}}{{\bar{c}}^{3}}-\frac{{\bar{c}}_{\xi \xi }}{\bar{c}}+\frac{{\bar{c}}_{\xi }^{2}}{{\bar{c}}^{2}}) \end{array} \end{array}\right),\\ t & =\left(\begin{array}{cc} \begin{array}{l} v_{G}(\bar{c}{\bar{\theta }}_{\xi }+{\bar{c}}_{\xi }\bar{\theta }+\bar{c}{{}_{1}}_{,\xi }\bar{\theta }+2{\bar{c}}_{1}{\bar{\theta }}_{\xi })\\ \;+\;\delta_{G}\left(2\frac{{\bar{c}}_{1}{\bar{c}}_{\xi }}{{\bar{c}}^{2}}-\frac{{\bar{c}}_{\xi }}{\bar{c}}-\frac{\bar{c}{{}_{1}}_{,\xi }}{\bar{c}}\right) \end{array} & \begin{array}{l} v_{G}\phi (\bar{c}{{}_{1}}_{,\xi }\bar{\theta }+2{\bar{c}}_{1}{\bar{\theta }}_{\xi })\\ \;+\;\delta_{G}\phi \left(2\frac{{\bar{c}}_{1}{\bar{c}}_{\xi }}{{\bar{c}}^{2}}-\frac{\bar{c}{{}_{1}}_{,\xi }}{\bar{c}}\right) \end{array}\\ \begin{array}{l} v_{G}({\bar{c_{2}}}_{,\xi }\bar{\theta }+2{\bar{c}}_{2}{\bar{\theta }}_{\xi })\\ \;+\;\delta_{G}\left(2\frac{{\bar{c}}_{2}{\bar{c}}_{\xi }}{{\bar{c}}^{2}}-\frac{\bar{c}{{}_{2}}_{,\xi }}{\bar{c}}\right) \end{array} & \begin{array}{l} v_{G}(\bar{c}{\bar{\theta }}_{\xi }+{\bar{c}}_{\xi }\bar{\theta }+\phi \bar{c}{{}_{2}}_{,\xi }\bar{\theta }+2\phi {\bar{c}}_{2}{\bar{\theta }}_{\xi })\\ \;+\;\delta_{G}\left(2\phi \frac{{\bar{c}}_{2}{\bar{c}}_{\xi }}{{\bar{c}}^{2}}-\frac{{\bar{c}}_{\xi }}{\bar{c}}-\phi \frac{\bar{c}{{}_{2}}_{,\xi }}{\bar{c}}\right) \end{array} \end{array}\right)\\ \;\text{and}\;d & =\left(\begin{array}{cc} v_{G}{\bar{c}}_{1}\bar{\theta }+\delta_{G}\left(-\frac{{\bar{c}}_{1}}{\bar{c}}+1\right) & v_{G}\phi {\bar{c}}_{1}\bar{\theta }+\delta_{G}\phi \left(-\frac{{\bar{c}}_{1}}{\bar{c}}\right)\\ v_{G}{\bar{c}}_{2}\bar{\theta }+\delta_{G}\left(-\frac{{\bar{c}}_{2}}{\bar{c}}\right) & v_{G}\phi {\bar{c}}_{2}\bar{\theta }+\delta_{G}\left(-\frac{\phi {\bar{c}}_{2}}{\bar{c}}+1\right) \end{array}\right) \end{array}$$

are the relaxation, transport and diffusion matrices, respectively.

To study the evolution of the fluctuated concentrations of each of the components, the following quantities are introduced:

$$\hat{c}={\hat{c}}_{1}+\phi {\hat{c}}_{2},$$

where $\hat{c}$ is the fluctuation in the total concentration, ${\hat{c}}_{1}$ is the fluctuation in vapour concentration and ${\hat{c}}_{2}$ is the fluctuation in air concentration. After some algebra and simplification, the equation for total fluctuated concentration is given by

3.2 $$\frac{\partial \hat{c}}{\partial \tau }=v_{G}\frac{\partial }{\partial \xi }{\left((\hat{c}\bar{\theta }\right)}_{\xi }\bar{c}+{\left(\bar{c}\bar{\theta }\right)}_{\xi }\hat{c})-\frac{\beta_{G}}{\epsilon }A\sqrt{\bar{\theta }}{\hat{c}}_{1}.$$

### 3.1. Boundary conditions

The boundary conditions depend on the setting of the problem. A profile at steady state is accompanied by its own set of boundary conditions depending on the conditions of the surroundings. As far as the fluctuations are concerned, Neumann boundary conditions are used. Here, care is taken to prevent the accumulation of fluctuations in the system. Hence, at the inner boundary (*ξ*=0),

3.3 $$\frac{\partial {\hat{c}}_{1}}{\partial \xi }(0,\tau )=0\;\text{and}\;\frac{\partial {\hat{c}}_{2}}{\partial \xi }(0,\tau )=0,$$

and at the outer boundary (*ξ*=1),

3.4 $$\frac{\partial {\hat{c}}_{1}}{\partial \xi }(1,\tau )=0\;\text{and}\;\frac{\partial {\hat{c}}_{2}}{\partial \xi }(1,\tau )=0$$

are taken.

### 3.2. Theoretical analysis

It can be observed that the evolution involves relaxation, transport and diffusion. According to definition 3.1, the system of equations (3.1) is well-posed provided the diffusion matrix, *d*, is positive definite.

**Definition 3.1 [15].**

Let

3.5 $${\text{v}}_{\tau }(\tau ,\xi )=R(\xi )\text{v}(\tau ,\xi )+T(\xi ){\text{v}}_{\xi }(\tau ,\xi )+D(\xi ){\text{v}}_{\xi \xi }(\tau ,\xi )$$

represent the Petrovskii parabolic equation (3.1). The initial value problem for the system (3.6) is well-posed if for any time *T*≥0, there is a constant *K*_*T*_ such that any solution $\text{ v}{\left(\xi ,\tau )=(\begin{array}{c} {\hat{c}}_{1}\\ {\hat{c}}_{2} \end{array}\right)}_{\tau }$ satisfies

3.6 $$\int_{-\infty }^{\infty }|\text{v}(\xi ,\tau ){|}^{2}\;d\xi \leq K_{T}\int_{-\infty }^{\infty }|\text{v}(\xi ,0){|}^{2}\;d\xi ,$$

for 0≤*τ*≤*T*.

Boundary conditions are said to be well-posed if the solution of the partial differential equation depends continuously on the boundary data [15]. Neumann boundary conditions are known to satisfy these criteria.

Let *D* be the diffusion matrix in (3.1). For the problem to be well-posed, the matrix

3.7 $$D_{\mathrm{sym}}=\left(\begin{array}{cc} v_{G}{\bar{c}}_{1}\bar{\theta }+\delta_{G}-\delta_{G}\frac{{\bar{c}}_{1}}{\bar{c}} & \frac{1}{2}\left(v_{G}\phi {\bar{c}}_{1}\bar{\theta }-\delta_{G}\phi \frac{{\bar{c}}_{1}}{\bar{c}}+v_{G}{\bar{c}}_{2}\bar{\theta }-\delta_{G}\frac{{\bar{c}}_{2}}{\bar{c}}\right)\\ \frac{1}{2}\left(v_{G}\phi {\bar{c}}_{1}\bar{\theta }-\delta_{G}\phi \frac{{\bar{c}}_{1}}{\bar{c}}+v_{G}{\bar{c}}_{2}\bar{\theta }-\delta_{G}\frac{{\bar{c}}_{2}}{\bar{c}}\right) & v_{G}\phi {\bar{c}}_{2}\bar{\theta }+\delta_{G}-\delta_{G}\phi \frac{{\bar{c}}_{2}}{\bar{c}} \end{array}\right),$$

where $D_{\mathrm{sym}}=\frac{1}{2}(D+D^{T})$ should be positive definite. Therefore, the well-posedness of the problem heavily depends on the values of $\bar{c}$, ${\bar{c}}_{1}$, ${\bar{c}}_{2}$ and $\bar{\theta }$.

**Theorem 3.2.**

*The system (3.1) is well-posed if
*

$$v_{G}\delta_{G}\bar{c}\bar{\theta }>\frac{{\left(v_{G}\bar{\theta }-\delta_{G}/\bar{c}\right)}^{2}{\left({\bar{c}}_{1}\phi -{\bar{c}}_{2}\right)}^{2}}{4}.$$

**Proof.**

Firstly, the trace of (3.7) which is given by the sum of its diagonal elements is calculated.

3.8 $$\begin{array}{rl} \mathrm{Tr}(D_{\mathrm{sym}}) & =\sum_{i=1}^{2}{D_{\mathrm{sym}}}_{ii}\\ & =\left(v_{G}{\bar{c}}_{1}\bar{\theta }+\delta_{G}-\delta_{G}\frac{{\bar{c}}_{1}}{\bar{c}}\right)+\left(v_{G}\phi {\bar{c}}_{2}\bar{\theta }+\delta_{G}-\delta_{G}\phi \frac{{\bar{c}}_{2}}{\bar{c}}\right)\\ & =v_{G}{\bar{c}}_{1}\bar{\theta }+\delta_{G}\\ & >0. \end{array}$$

From (3.8), it can be deduced that *D*_sym_ is semi-positive definite. Hence, it is important to prove that its determinant is positive to ensure the positive definiteness of (3.7) and thus the well-posedness of (3.1).

The determinant of *D*_sym_ is given by

3.9 $$\begin{array}{rl} \mathrm{Det}(D_{\mathrm{sym}}) & =-\frac{1}{4{\bar{c}}^{2}}({v_{G}}^{2}\phi^{2}{\bar{\theta }}^{2}{\bar{c}}^{2}{\bar{c}}_{1}^{2}-2{v_{G}}^{2}\phi {\bar{\theta }}^{2}{\bar{c}}^{2}{\bar{c}}_{1}{\bar{c}}_{2}+{v_{G}}^{2}{\bar{\theta }}^{2}{\bar{c}}^{2}{\bar{c}}_{2}^{2}\\ & \;-2v_{G}\delta_{G}\phi^{2}\bar{\theta }\bar{c}{\bar{c}}_{1}^{2}-4v_{G}\delta_{G}\phi \bar{\theta }{\bar{c}}^{2}{\bar{c}}_{2}+4v_{G}\delta_{G}\phi \bar{\theta }\bar{c}{\bar{c}}_{1}{\bar{c}}_{2}\\ & \;-4v_{G}\delta_{G}\bar{\theta }{\bar{c}}^{2}{\bar{c}}_{1}-2v_{G}\delta_{G}\bar{\theta }\bar{c}{\bar{c}}_{2}^{2}+{\delta_{G}}^{2}\phi^{2}{\bar{c}}_{1}^{2}+4{\delta_{G}}^{2}\phi \bar{c}{\bar{c}}_{2}\\ & \;-2{\delta_{G}}^{2}\phi {\bar{c}}_{1}{\bar{c}}_{2}-4{\delta_{G}}^{2}{\bar{c}}^{2}+4{\delta_{G}}^{2}\bar{c}{\bar{c}}_{1}+{\delta_{G}}^{2}{\bar{c}}_{2}^{2}). \end{array}$$

Upon simplifying (3.9), the following is obtained:

3.10 $$Det(D_{\mathrm{sym}})=-\frac{1}{4}{\left(v_{G}\bar{\theta }-\frac{\delta_{G}}{\bar{c}}\right)}^{2}{\left(\phi {\bar{c}}_{1}-{\bar{c}}_{2}\right)}^{2}+v_{G}\delta_{G}\bar{c}\bar{\theta },$$

where *v*_*G*_>0, *δ*_*G*_>0 and *ϕ*>0. The only varying terms will be the concentrations and temperature at steady state.

Clearly, from (3.10) for *Det*(*D*_sym_)>0,

$$v_{G}\delta_{G}\bar{c}\bar{\theta }>\frac{1}{4}{\left(v_{G}\bar{\theta }-\frac{\delta_{G}}{\bar{c}}\right)}^{2}{\left({\bar{c}}_{1}\phi -{\bar{c}}_{2}\right)}^{2},$$

which proves the Theorem. ▪

By definition, a well-posed problem is one where a solution exists, the solution is unique and the solution depends continuously on the data. However, this is rarely achieved in applied mathematical problems [16]. The problem is influenced by experimental values which are only measured at a discrete number of points. Therefore, the problem cannot depend continuously on the data, and hence becomes ill-posed. A suitable choice of initial and boundary conditions can remedy the ill-posed nature of the problem.

Next, the three main phenomena involved in the evolution of air and vapour concentration, that is, relaxation, transport and diffusion, are discussed. These processes are examined using the principle of superposition. If the sum of these three functions is a solution of a linear PDE, then by the principle of superposition, each of the functions is individually also a solution of the PDE (http://www.owlnet.rice.edu/∼ceng501/Chap7.pdf (accessed 11 May 2017)).

### 3.3. Relaxation, transport and diffusion

The relaxation effect of ${\hat{c}}_{1}$ on $\hat{c}{{}_{1}}_{\tau }$ and ${\hat{c}}_{2}$ on $\hat{c}{{}_{2}}_{\tau }$ is governed by the equations below:

3.11 $$\frac{\partial {\hat{c}}_{1}}{\partial \tau }=r_{11}{\hat{c}}_{1}$$

and

3.12 $$\frac{\partial {\hat{c}}_{2}}{\partial \tau }=r_{22}{\hat{c}}_{2},$$

and the solutions are

$${\hat{c}}_{1}=a_{1}\;\exp(r_{11}\tau )\;\text{and}\;{\hat{c}}_{2}=a_{2}\;\exp(r_{22}\tau ),$$

where *a*_1_ and *a*_2_ are constants, respectively.

Irrespective of the signs of the constants *a*_1_ and *a*_2_, if *r*_11_<0 and *r*_22_<0, (3.11) and (3.12) bring about natural relaxation. ${\hat{c}}_{1}$ and ${\hat{c}}_{2}$ tend towards zero, as required. When *r*_11_>0 and *r*_22_>0, the solution ${\hat{c}}_{1}$ can increase infinitely and thus become unbounded. However, diffusion present in the system given by (3.1) damps the increasing relaxation term, as shown later.

The cross effect of ${\hat{c}}_{2}$ on the evolution of ${\hat{c}}_{1}$, and vice versa, is now studied. Consider the following equations:

3.13 $$\frac{\partial {\hat{c}}_{1}}{\partial \tau }=r_{12}{\hat{c}}_{2}$$

and

3.14 $$\frac{\partial {\hat{c}}_{2}}{\partial \tau }=r_{21}{\hat{c}}_{1}.$$

With *r*_12_<0 and *r*_21_<0, it is indicated that an increase in ${\hat{c}}_{2}$ results in a decrease in $\hat{c}{{}_{1}}_{\tau }$, whereas decreasing ${\hat{c}}_{2}$ increases $\hat{c}{{}_{1}}_{\tau }$. A similar observation is true for (3.14). On the other hand, if *r*_12_>0 and *r*_21_>0, an increase or decrease on the r.h.s. of (3.13) and (3.14) directly increases or decreases, respectively, ${\hat{c}}_{1}$ and ${\hat{c}}_{2}$.

A relaxing system causes the fluctuations to die out with time. In this situation, the wearer experiences better comfort due to the presence of a steady amount of vapour and air in the batting. Increasing amplitude of fluctuations mean the presence of more air/vapour in the fabric, which results in discomfort.

The following equations:

3.15 $$\frac{\partial {\hat{c}}_{1}}{\partial \tau }=t_{11}\frac{\partial {\hat{c}}_{1}}{\partial \xi }$$

and

3.16 $$\frac{\partial {\hat{c}}_{2}}{\partial \tau }=t_{22}\frac{\partial {\hat{c}}_{2}}{\partial \xi }$$

are pure advection equations. They do not cause any amplification or decay of the solution. These equations solely transport the fluctuations towards or away from the skin, depending on the signs of the coefficients. *t*_11_>0 and *t*_22_>0 take the fluctuations near the external environment, and if *t*_11_<0 and *t*_22_<0, then fluctuations are advected closer to the human body. ${\hat{c}}_{1}$

and ${\hat{c}}_{2}$ have a mutual influence on the transportation of each other. This can be seen in (3.17) and (3.18):

3.17 $$\frac{\partial {\hat{c}}_{1}}{\partial \tau }=t_{12}\frac{\partial {\hat{c}}_{2}}{\partial \xi }$$

and

3.18 $$\frac{\partial {\hat{c}}_{2}}{\partial \tau }=t_{21}\frac{\partial {\hat{c}}_{1}}{\partial \xi }.$$

Equations (3.17) and (3.18) give

3.19 $$\frac{\partial^{2}{\hat{c}}_{1}}{\partial \tau^{2}}=t_{12}t_{21}\frac{\partial^{2}{\hat{c}}_{1}}{\partial \xi^{2}}.$$

Equation (3.19) indicates that, for *t*_12_*t*_21_>0, diffusion is added to the advection of ${\hat{c}}_{1}$; otherwise anti-diffusion is included. The same findings hold for ${\hat{c}}_{2}$.

The pure diffusion equations are as follows:

3.20 $$\frac{\partial {\hat{c}}_{1}}{\partial \tau }=d_{11}\frac{\partial^{2}{\hat{c}}_{1}}{\partial \xi^{2}}$$

and

3.21 $$\frac{\partial {\hat{c}}_{2}}{\partial \tau }=d_{22}\frac{\partial^{2}{\hat{c}}_{2}}{\partial \xi^{2}}.$$

Positive diffusion coefficients dissipate the fluctuations whereas negative coefficients act as anti-diffusion terms. It can be seen in the following equations that ${\hat{c}}_{1}$ is affected by the diffusive nature of ${\hat{c}}_{2}$ and vice versa. The concerned equations are

3.22 $$\frac{\partial {\hat{c}}_{1}}{\partial \tau }=d_{12}\frac{\partial^{2}{\hat{c}}_{2}}{\partial \xi^{2}}$$

and

3.23 $$\frac{\partial {\hat{c}}_{2}}{\partial \tau }=d_{21}\frac{\partial^{2}{\hat{c}}_{1}}{\partial \xi^{2}}.$$

Differentiating (3.22) with respect to *τ*, the following is obtained:

3.24 $$\frac{\partial^{2}{\hat{c}}_{1}}{\partial \tau^{2}}=d_{12}{\left(\frac{\partial {\hat{c}}_{2}}{\partial \tau }\right)}_{\xi \xi }.$$

Equation (3.24) can be written as

3.25 $$\frac{\partial^{2}{\hat{c}}_{1}}{\partial \tau^{2}}=d_{12}d_{21}\frac{\partial^{4}{\hat{c}}_{1}}{\partial \xi^{4}}.$$

The last term in equation (3.25) adds or reduces the dissipation property of the system depending on the terms *d*_12_ and *d*_21_. The same holds for the influence of $\hat{c}{{}_{1}}_{\xi \xi }$ on $\hat{c}{{}_{2}}_{\tau }$.

As pointed out earlier, this dissipative nature of the system has a major role in the boundedness of the system. Positive diffusion coefficients for the problem in 0≤*ξ*≤1 can ensure that the system (3.1) remains bounded and stable. This can be seen through the energy estimate of the diffusion process. Consider the following coupled diffusion equations:

3.26 $$\hat{c}{{}_{1}}_{\tau }=d_{11}\Delta {\hat{c}}_{1}+d_{12}\Delta {\hat{c}}_{2}$$

and

3.27 $$\hat{c}{{}_{2}}_{\tau }=d_{21}\Delta {\hat{c}}_{1}+d_{22}\Delta {\hat{c}}_{2}.$$

The square of the *L*^2^-norm on ${\hat{c}}_{1}$ is defined as

3.28 $$∥{\hat{c}}_{1}{∥}^{2}=\int_{0}^{1}{\hat{c}}_{1}^{2}\;d\xi .$$

From (3.26) and (3.28),

3.29 $$\begin{array}{rl} \frac{1}{2}\frac{d}{d\tau }∥{\hat{c}}_{1}{∥}^{2} & =\frac{1}{2}\frac{d}{d\tau }\int_{0}^{1}{\hat{c}}_{1}^{2}\;d\xi \\ & =\int_{0}^{1}{\hat{c}}_{1}\hat{c}{{}_{1}}_{\tau }\;d\xi \\ & =-d_{11}∥\nabla {\hat{c}}_{1}{∥}^{2}-d_{12}\langle \nabla {\hat{c}}_{1}\nabla {\hat{c}}_{2}\rangle . \end{array}$$

Similarly, it is easily seen that

3.30 $$\frac{1}{2}\frac{d}{d\tau }∥{\hat{c}}_{2}{∥}^{2}=-d_{21}\langle \nabla {\hat{c}}_{1}\nabla {\hat{c}}_{2}\rangle -d_{22}∥\nabla {\hat{c}}_{2}{∥}^{2}.$$

The energy, *E*(*τ*) is defined as follows:

3.31 $$E(\tau )=\frac{1}{2}∥{\hat{c}}_{1}{∥}^{2}+\frac{\lambda }{2}∥{\hat{c}}_{2}{∥}^{2},$$

where λ>0 is a coupling parameter chosen so as to obtain the most appropriate stability result [17].

The energy equation for the coupled equations (3.26) and (3.27) is thus given by

3.32 $$\frac{dE}{d\tau }=-d_{11}∥\nabla {\hat{c}}_{1}{∥}^{2}-\lambda d_{22}∥\nabla {\hat{c}}_{2}{∥}^{2}-(d_{12}+\lambda d_{21})\langle \nabla {\hat{c}}_{1}\nabla {\hat{c}}_{2}\rangle .$$

Using the Poincaré inequality [17], (3.32) can be written as

3.33 $$\frac{dE}{d\tau }\leq -E(\tau )(e_{1}+e_{2}),$$

where

$$e_{1}=\max(d_{12},d_{21})\frac{\pi^{2}}{2}$$

and

$$e_{2}=\max\;\left(\frac{\pi^{2}(d_{12}+\lambda d_{21})\langle {\hat{c}}_{1}{\hat{c}}_{2}\rangle }{E(\tau )}\right).$$

The following result is thus obtained:

3.34 $$\frac{dE}{d\tau }\leq -\omega E(\tau ),$$

that is,

3.35 E(τ)≤E(0) exp⁡(−ωτ),

where *ω*=*e*_1_+*e*_2_>0. As a result, the diffusion equations are monotone decreasing.

Clearly, the role of these phenomena depend on the system (3.1) and the steady-state scenario.

### 3.4. Qualitative properties of the relaxation–diffusion system

Each phenomenon has a distinct role in the evolution of the fluctuations. However, the transport phenomenon is the least dominant one. This can be seen through the Peclet number [18], which is very small, as given in the following:

3.36 $$Pe=\frac{|v_{G}|l\tau_{c}}{\epsilon \delta_{G}},$$

where *l* is the mean diameter of the pores. The same is confirmed by the numerical experiments in §4. As a result, the transport term will not affect the solution of (3.1) significantly. This permits the omission of the advection term in the analysis conducted under this section.

Consider the relaxation–diffusion equation

3.37 $$\left\{ \begin{array}{ll} {\text{v}}_{\tau }-d^{-}{\text{v}}_{\xi \xi }=r^{+}\text{v} & \text{a.e in}\;\Omega \times ]0,\tau_{\mathrm{final}}[,\\ {\text{v}}_{\xi }=0 & \text{a.e in}\;\partial \Omega \times ]0,\tau_{\mathrm{final}}[\\ \text{v}(\xi ,0)={\text{v}}_{0}(\xi ) & \text{a.e in}\;\partial \Omega , \end{array}\right.$$

where $\text{v}=(\begin{array}{c} {\hat{c}}_{1}\\ {\hat{c}}_{2} \end{array})$, $r^{+}=\max(r(\xi ))$ for *r*(*ξ*)≤0 and $d^{-}=\min(d(\xi ))$ for *d*(*ξ*)≥0. Let *Ω* denote the domain such that 0≤*ξ*≤1 and *τ*_final_ be such that 0<*τ*<*τ*_final_.

Now, the variational formulation of the problem given by (3.37) is derived. The idea of a variational formulation is to express (3.37) as a second-order ODE problem amenable to an existence and uniqueness theory.

Multiplying (3.37) by a test function *ν*(*ξ*), which does not depend on the time *τ*, and integrating with respect to *ξ* considering the boundary conditions, the following equation is obtained:

3.38 $$\int_{\Omega }\frac{\partial \text{v}(\xi ,\tau )}{\partial \tau }\nu (\xi )\;d\xi +d^{-}\int_{\Omega }\nabla \text{v}(\xi ,\tau )\cdot \nabla \nu (\xi )\;d\xi =r^{+}\int_{\Omega }\text{v}(\xi ,\tau )\nu (\xi )\;d\xi .$$

As neither *Ω* nor *ν*(*ξ*) depend on *τ*, (3.38) can be rewritten as

3.39 $$\frac{d}{d\tau }\int_{\Omega }\text{v}(\xi ,\tau )\nu (\xi )\;d\xi +d^{-}\int_{\Omega }\nabla \text{v}(\xi ,\tau )\cdot \nabla \nu (\xi )\;d\xi =r^{+}\int_{\Omega }\text{v}(\xi ,\tau )\nu (\xi )\;d\xi .$$

Owing to the fact that the variables *ξ* and *τ* have very distinct roles, they can be separated considering the solution **v**(*ξ*,*τ*) as a function of time that is evaluated on a functional space defined on *Ω*. Thus, if the final time *τ*_final_>0, **v**(*ξ*,*τ*) is defined by

$$\begin{array}{rl} \text{v}:\;]0,\tau_{\mathrm{final}}[ & \to H^{1}(\Omega )\\ \tau & \to \text{v}(\tau ) \end{array}$$

and **v**(*ξ*,*τ*) still takes the value **v**(*τ*)(*ξ*).

Next, the scalar product *L*^2^(*Ω*) and bilinear *a*(*ω*,*ν*) are defined as

3.40 $${\left\langle \omega ,\nu \right\rangle }_{L^{2}(\Omega )}=\int_{\Omega }\omega (\xi )\nu (\xi )\;d\xi$$

and

3.41 $$a(\omega ,\nu )=\int_{\Omega }\nabla \omega (\xi )\cdot \nabla \nu (\xi )\;d\xi .$$

The variational formulation of (3.38), for *ν*(*ξ*)∈*H*^1^(*Ω*), is given as follows:

3.42 $$\left\{ \begin{array}{l} \frac{d}{d\tau }{\left\langle \text{v}(\tau ),\nu \right\rangle }_{L^{2}(\Omega )}+d^{-}\;a(\text{v}(\tau ),\nu )=r^{+}{\left\langle \text{v}(\tau ),\nu \right\rangle }_{L^{2}(\Omega ),}\;\forall \nu \in H^{1}(\Omega ),\;0<\tau <\tau_{\mathrm{final}}\\ \text{v}(\tau =0)={\text{v}}_{0}. \end{array}\right.$$

In the section that follows, it is established that the solution of (3.42) exists and is unique.

#### 3.4.1. Existence and uniqueness

**Theorem 3.3 [19].**

*Let H*^1^*(Ω) and L*^2^*(Ω) be two real Hilbert spaces with infinite dimension. Suppose that H*^1^*(Ω)⊂L*^2^*(Ω) with a compact injection and that H*^1^*(Ω) is dense in L*^2^*(Ω). Let a(⋅,⋅) be continuous symmetric bilinear and coercive in H*^1^*(Ω). Then the eigenvalues of* $a(\text{v},\nu )=\tilde{\lambda }{\left\langle \text{v},\nu \right\rangle }_{L^{2}(\Omega )}$*, ∀ν∈H*^1^*(Ω) and* $\tilde{\lambda }\in R$ *form an increasing positive real sequence* ${\left({\tilde{\lambda }}_{h}\right)}_{h\geq 1}$ *which tends to infinity. There exists a Hilbert’s basis of L*^2^(*Ω*) (**v**_*h*_)_*h*≥1_ *of associated eigenvectors, that is,*

$${\text{v}}_{h}\in H^{1}(\Omega ),\;\text{and}\;\;a({\text{v}}_{h},\nu )={\tilde{\lambda }}_{h}{\left\langle {\text{v}}_{h},\nu \right\rangle }_{L^{2}(\Omega )}\;\forall \nu \in H^{1}(\Omega ).$$

**Proposition 3.4.**

*Let H*^1^(*Ω*) *and L*^2^(*Ω*) *be Hilbert spaces such that H*^1^(*Ω*)⊂*L*^2^(*Ω*) *with a compact injection and H*^1^(*Ω*) *is dense in L*^2^(*Ω*). *Let a*(*ω*,*ν*) *be continuous symmetric bilinear and coercive in H*^1^(*Ω*). *Given that τ*_final_>0 and **v**_0_∈*L*^2^(*Ω*), *the problem (3.42) has a unique solution* **v**∈*C*([0,*τ*_final_];*L*^2^(*Ω*))∩*L*^2^(]0,*τ*_final_[;*H*^1^(*Ω*)).

**Proof.**

The first part of the proof assumes the existence of a solution **v**. An explicit form of **v** in terms of a series is obtained from the spectral decomposition of *H*^1^(*Ω*) and *L*^2^(*Ω*), which proves the uniqueness of the solution **v**. The next part shows that this series converges in *L*^2^(]0,*τ*_final_[;*H*^1^(*Ω*)) and *C*([0,*τ*_final_];*L*^2^(*Ω*)), and the sum is a solution of (3.42).

Let **v**∈*C*([0,*τ*_final_];*L*^2^(*Ω*))∩*L*^2^(]0,*τ*_final_[;*H*^1^(*Ω*)) be a solution of (3.42). Define

3.43 $$\alpha_{h}(\tau )={\left\langle \text{v}(\tau ),{\text{v}}_{h}\right\rangle }_{L^{2}(\Omega )},\;\alpha_{h}^{0}={\left\langle {\text{v}}_{0},{\text{v}}_{h}\right\rangle }_{L^{2}(\Omega )},\;\text{for}\;\alpha_{h}(\tau )\in C([0,\tau_{\mathrm{final}}]).$$

As (**v**_*h*_)_*h*≥1_ is a Hilbert’s basis in *L*^2^(*Ω*),

3.44 $$\text{v}(\tau )=\sum_{h=1}^{+\infty }\alpha_{h}(\tau ){\text{v}}_{h}.$$

Taking *ν*=**v**_*h*_ in (3.42) and by theorem 3.3, the following is obtained:

3.45 $$\left\{ \begin{array}{l} \frac{d\alpha_{h}}{d\tau }+d^{-}{\bar{\lambda }}_{h}\alpha_{h}=r^{+}\alpha_{h}\;\text{in}\;]0,\tau_{\mathrm{final}}[\\ \alpha_{h}(\tau =0)=\alpha_{h}^{0}. \end{array}\right.$$

The unique solution of (3.45) given by

3.46 $$\alpha_{h}(\tau )=\alpha_{h}^{0}\exp(-(d^{-}{\bar{\lambda }}_{h}-r^{+})\tau )\;\text{for}\;\tau >0$$

is an explicit formula for the solution **v**.

It remains to show that the series

3.47 $$\sum_{i=1}^{+\infty }(\alpha_{i}^{0}\exp(-(d^{-}{\bar{\lambda }}_{i}-r^{+})\tau )){\text{v}}_{i}$$

converges in *C*([0,*τ*_final_];*L*^2^(*Ω*))∩*L*^2^(]0,*τ*_final_[;*H*^1^(*Ω*)), and that its sum, **v**(*τ*), is a solution of (3.42).

Consider the partial sum of order *h* of the series (3.47)

3.48 $$\varphi^{h}(\tau )=\sum_{i=1}^{h}(\alpha_{i}^{0}\exp(-(d^{-}{\bar{\lambda }}_{i}-r^{+})\tau )){\text{v}}_{i}.$$

We have *φ*^*h*^∈*C*([0,*τ*_final_];*L*^2^(*Ω*)) because each *α*_*i*_(*τ*) is continuous.

For *m*>*h*, using the orthonormal property of the eigenfunction **v**_*i*_, the following results:

3.49 $$\begin{array}{rl} ∥\varphi^{m}(\tau )-\varphi^{h}(\tau ){∥}_{L^{2}(\Omega )} & ={∥\sum_{i=h+1}^{m}\alpha_{i}^{0}\;\exp(-(d^{-}{\bar{\lambda }}_{i}-r^{+})\tau ){\text{v}}_{i}∥}_{L^{2}(\Omega )}\\ & \leq {\left(\sum_{i=h+1}^{m}|\alpha_{i}^{0}{|}^{2}\exp(-2(d^{-}{\bar{\lambda }}_{i}-r^{+})\tau ){\text{v}}_{i}\right)}^{1/2}\\ & \leq {\left(\sum_{i=h+1}^{m}|\alpha_{i}^{0}{|}^{2}\right)}^{1/2}, \end{array}$$

provided $d^{-}{\bar{\lambda }}_{i}-r^{+}\geq 0$ and because the eigenvalues $\left({\bar{\lambda }}_{i}\right)$ have a strictly positive increasing sequence.

As **v**_0_∈*L*^2^(*Ω*),

3.50 $$∥{\text{v}}_{0}{∥}_{L^{2}(\Omega )}^{2}=\sum_{i=1}^{+\infty }|\alpha_{i}^{0}{|}^{2}<+\infty$$

leads to the fact that the series *φ*^*h*^(*τ*) is Cauchy in *L*^2^(*Ω*). Additionally, it can be deduced that *φ*^*h*^ verifies

3.51 $$\lim_{h,m\to +\infty }\left(\underset{0\leq \tau \leq \tau_{\mathrm{final}}}{\sup}∥\varphi^{m}(\tau )-\varphi^{h}(\tau ){∥}_{L^{2}(\Omega )}\right)=0,$$

that is, it is Cauchy in *C*([0,*τ*_final_];*L*^2^(*Ω*)).

Next, for *m*>*h*,

3.52 $$\begin{array}{rl} ∥\varphi^{m}(\tau )-\varphi^{h}(\tau ){∥}_{H^{1}(\Omega )}^{2} & =a(\varphi^{m}(\tau )-\varphi^{h}(\tau ),\varphi^{m}(\tau )-\varphi^{h}(\tau ))\\ & =\sum_{i=h+1}^{m}{\bar{\lambda }}_{i}|\alpha_{i}(\tau ){|}^{2}\\ & \leq 2\sum_{i=h+1}^{m}{\bar{\lambda }}_{i}|\alpha_{i}^{0}{|}^{2}\exp(-2(d^{-}{\bar{\lambda }}_{i}-r^{+})\tau ). \end{array}$$

Hence,

3.53 $$\int_{0}^{\tau_{\mathrm{final}}}∥\varphi^{m}(\tau )-\varphi^{h}(\tau ){∥}_{H^{1}(\Omega )}^{2}\;d\tau \leq \sum_{i=h+1}^{m}|\alpha_{i}^{0}{|}^{2},$$

provided $d^{-}{\bar{\lambda }}_{i}-r^{+}\geq 0$. This implies that the series *φ*^*h*^ satisfies

3.54 $$\lim_{h,m\to +\infty }\int_{0}^{\tau_{\mathrm{final}}}∥\varphi^{m}(\tau )-\varphi^{h}(\tau ){∥}_{H^{1}(\Omega )}^{2}\;d\tau =0,$$

which means that it is Cauchy in *L*^2^(]0,*τ*_final_[;*H*^1^(*Ω*)).

As both *C*([0,*τ*_final_];*L*^2^(*Ω*)) and *L*^2^(]0,*τ*_final_[;*H*^1^(*Ω*)) are complete spaces, the Cauchy series *φ*^*h*^ converges and its limit **v** is defined as

3.55 $$\lim_{h\to +\infty }\varphi^{h}=\text{v}\;\text{in}\;C([0,\tau_{\mathrm{final}}];L^{2}(\Omega ))\cap L^{2}(]0,\tau_{\mathrm{final}}[;H^{1}(\Omega )).$$

The fact that *φ*^*h*^(0) converges to **v**_0_ in *L*^2^(*Ω*), the desired initial condition **v**(0)=**v**_0_ can be deduced. Clearly, **v**(*τ*) being the sum of the series (3.47) implies that it also satisfies the variational formulation (3.42) for every test function *ν*=**v**_*h*_. As a result, **v**(*τ*) is a solution of (3.42). ▪

The positivity of the solution is given by proposition 3.5, which can be stated as follows.

#### 3.4.2. Positivity

**Proposition 3.5**

*Let Ω be an open bounded space in* $R^{N}$, *and τ*_final_>0. *Let* **v**_0_∈*L*^2^(*Ω*) *and* **v**∈*C*([0,*τ*_final_];*L*^2^(*Ω*))∩*L*^2^(]0,*τ*_final_[;*H*^1^(*Ω*)) *be a unique solution of* (3.37). *If* **v**_0_≥0 *almost everywhere in Ω, then* **v**≥0 *a.e in* ]0,*τ*_final_[×*Ω*.

**Proof.**

Let ${\text{v}}^{-}=\min(\text{v},0)$ belonging to *L*^2^(]0,*τ*_final_[;*H*^1^(*Ω*)). Then, for 0<*τ*<*τ*_final_,

3.56 $$\nabla {\text{v}}^{-}=1_{\text{v}<0}\nabla \text{v}\;\text{a.e in}\;\Omega$$

and

3.57 $$\int_{\Omega }\nabla \text{v}(\tau )\cdot \nabla {\text{v}}^{-}(\tau )\;d\xi =\int_{\Omega }|\nabla {\text{v}}^{-}(\tau ){|}^{2}\;d\xi ,$$

where the function 1_**v**<0_(*ξ*)=1 at **v**(*ξ*)<0, and 0 elsewhere.

Similarly,

3.58 $$\int_{\Omega }\frac{\partial \text{v}(\tau )}{\partial \tau }{\text{v}}^{-}(\tau )\;d\xi =\frac{1}{2}\;\frac{d}{d\tau }\left(\int_{\Omega }|\nabla {\text{v}}^{-}(\tau ){|}^{2}\;d\xi \right).$$

Taking *ν*=**v**^−^ in the variational formulation (3.42), and using the identities (3.57) and (3.58) yields

3.59 $$\frac{1}{2}\frac{d}{d\tau }\int_{\Omega }|{\text{v}}^{-}{|}^{2}\;d\xi +d^{-}\int_{\Omega }|\nabla {\text{v}}^{-}{|}^{2}\;d\xi =r^{+}\int_{\Omega }|{\text{v}}^{-}{|}^{2}\;d\xi .$$

Integrating (3.59) with respect to time,

3.60 $$\frac{1}{2}\int_{\Omega }|{\text{v}}^{-}(\tau ){|}^{2}\;d\xi -\frac{1}{2}\int_{\Omega }|{\text{v}}^{-}(0){|}^{2}\;d\xi +d^{-}\int_{0}^{\tau }\int_{\Omega }|\nabla {\text{v}}^{-}{|}^{2}\;d\xi \;dw=r^{+}\int_{0}^{\tau }\int_{\Omega }|{\text{v}}^{-}{|}^{2}\;d\xi \;dw.$$

As **v**^−^(0)=(**v**_0_)^−^=0, for *r*^+^<0

3.61 $$\frac{1}{2}\int_{\Omega }|{\text{v}}^{-}(\tau ){|}^{2}\;d\xi +d^{-}\int_{0}^{\tau }\int_{\Omega }|\nabla {\text{v}}^{-}{|}^{2}d\xi \;dw\leq 0.$$

Hence, **v**^−^=0 a.e in ]0,*τ*_final_[×*Ω*, provided *d*^−^>0. ▪

#### 3.4.3. Verification of variational formulation

The result from proposition 3.4 is applied to the original PDE (3.37). The aim is to prove that the variational formulation can be solved for (3.37). This is done in proposition 3.8 below. Theorem 3.6 and proposition 3.7, respectively, which are quoted below, will be useful in order to establish the proof for proposition 3.8.

**Theorem 3.6 ((Trace Theorem) [19]).**

*Suppose that* $\Omega =R_{+}^{N}$*. The trace operator γ*_0_ *is defined as
*

$$\begin{array}{rl} H^{1}(\Omega )\cap C(\bar{\Omega }) & \to L^{2}(\partial \Omega )\cap C(\bar{\partial \Omega })\\ \nu & \to \gamma_{0}(\nu )=\nu {|}_{\partial \Omega }. \end{array}$$

*This operator γ*_0_ *is extended by continuity in a continuous linear application of H*^1^*(Ω) in L*^2^*(∂Ω). There exists a constant B>0 such that ∀ν∈H*^1^*(Ω),
*

$$∥\nu {∥}_{L^{2}(\partial \Omega )}\leq B∥\nu {∥}_{H^{1}(\Omega )}.$$

**Proposition 3.7 [19].**

*Let Ω be regular open bounded in* $R^{N}$, *and the final time τ*_final_>0. *For a given regular initial condition* **v**_0_∈*H*^1^(*Ω*), *the unique solution* **v**∈*C*([0,*τ*_final_];*L*^2^(*Ω*))∩*L*^2^(]0,*τ*_final_[;*H*^1^(*Ω*)) *of* (3.37) *is considered. Then, this solution is more regular in the sense* ∂**v**/∂*τ*∈*L*^2^(]0,*τ*_final_[;*L*^2^(*Ω*)) *and* **v**∈ *C*([0,*τ*_final_];*H*^1^(*Ω*))∩*L*^2^(]0,*τ*_final_[;*H*^2^(*Ω*)).

**Proposition 3.8.**

*Let Ω be open bounded in* $R^{N}$, *τ*_final_>0 *and* **v**_0_∈*L*^2^(*Ω*). *Then the parabolic PDE* (3.37) *has a unique solution* **v**∈*C*([0,*τ*_final_];*L*^2^(*Ω*))∩ *L*^2^(]0,*τ*_final_[;*H*^1^(*Ω*)).

**Proof.**

From proposition 3.4, the solution of variational formulation (3.42) of the PDE (3.37) admits a unique solution. It suffices to show that the unique solution **v**∈*C*([0,*τ*_final_];*L*^2^(*Ω*))∩*L*^2^(]0,*τ*_final_[;*H*^1^(*Ω*)) of this variational formulation is in fact the solution of (3.37).

The boundary conditions are obtained by the application of the Trace theorem 3.6 for **v**(*τ*)∈*H*^1^(*Ω*) for almost all *τ*∈]0,*τ*_final_[. The initial condition is justified by the continuity on **v**(*τ*) at *τ*=0.

If the solution **v** is sufficiently regular, which is true by proposition 3.7, then by integration by parts the variational formulation (3.42) is equivalent to

3.62 $$\int_{\Omega }\left(\frac{\partial \text{v}}{\partial \tau }-d^{-}\Delta \text{v}-r^{+}\text{v}\right)\nu \;d\xi =0,$$

∀*ν*∈*H*^1^(*Ω*) and almost all *τ*∈]0,*τ*_final_[. Consequently, from (3.62) it can be deduced that

3.63 $$\frac{\partial \text{v}}{\partial \tau }-d^{-}\Delta \text{v}-r^{+}\text{v}=0\;\text{a.e in}\;\Omega \times ]0,\tau_{\mathrm{final}}[.$$

 ▪

In the following section, the properties of (3.1) are verified numerically under three distinct situations, namely:

— *Case 1*. The environmental temperature and relative humidity are considered to be lower than that of the human body.
— *Case 2*. The relative humidity is taken to be higher in the environment compared to that of the body, while the same temperature profile as in Case 1 is maintained.
— *Case 3*. A constant temperature and relative humidity profile is taken from the internal to the external environments.

## 4. Numerical discussion and results

The numerical solution of (3.1) is sought through the semi-implicit Crank–Nicolson finite difference scheme. This numerical scheme gives second-order accuracy both in time and space, and is unconditionally stable. The results presented in this section have been generated in Matlab. Here, the phenomena discussed under three distinct situations are illustrated. The non-dimensional *τ* is taken such that 0≤*τ*≤1. A Gaussian profile

4.1 $${\hat{c}}_{1}(\xi ,0)={\hat{c}}_{2}(\xi ,0)=0.001\;\exp\;\left(-\frac{{\left(\xi -0.5\right)}^{2}}{0.01}\right)$$

is chosen as an initial configuration. This choice is simply made to confirm the behaviour of the fluctuations with respect to the different processes discussed in §3.3 and to compare the processes under three distinct cases. The initial condition is given by the solid black line in this section.

### 4.1. Case 1

**Figure 2.**
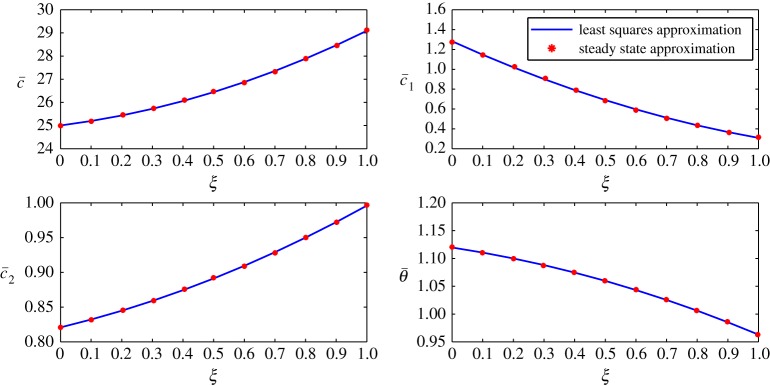
Least squares approximation of the parameters at steady state given by Huang *et al*. [10].

The steady-state profiles by Huang *et al*. [10] are interpolated by a least squares approximation and are given in figure 2. In this interpolation, 11 points are used from *ξ*=0 to *ξ*=1, with *Δξ*=0.1. The inner layer is exposed to the human body in contact with sweat in the form of vapour at approximately 303 K and higher humidity. The outer layer is in contact with cold moving air at approximately 253 K and lower humidity. In addition, there is no air supply at the inner cover. The equations are thus given by

4.2 $$\begin{array}{r} \bar{c}(\xi )=2.4103\xi^{2}+1.6746\xi +25.0041, \end{array}$$

4.3 $$\begin{array}{r} {\bar{c}}_{1}(\xi )=0.4231\xi^{2}-1.3966\xi +1.2825, \end{array}$$

4.4 $$\begin{array}{r} {\bar{c}}_{2}(\xi )=0.06896\xi^{2}+0.1062\xi +0.8208 \end{array}$$

4.5 $$\begin{array}{r} \;\text{and}\;\bar{\theta }(\xi )=-0.07331\xi^{2}-0.08321\xi +1.1196. \end{array}$$

The behaviour of each process under these conditions is summarized as follows:

#### 4.1.1. Relaxation process

**Figure 3.**
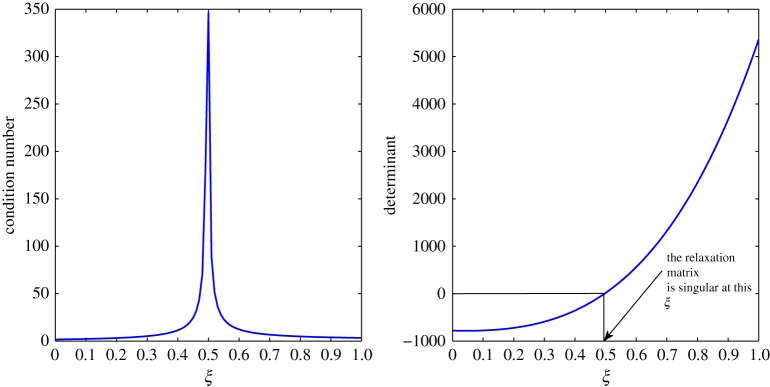
Condition number and determinant of the relaxation matrix in Case 1.

**Figure 4.**
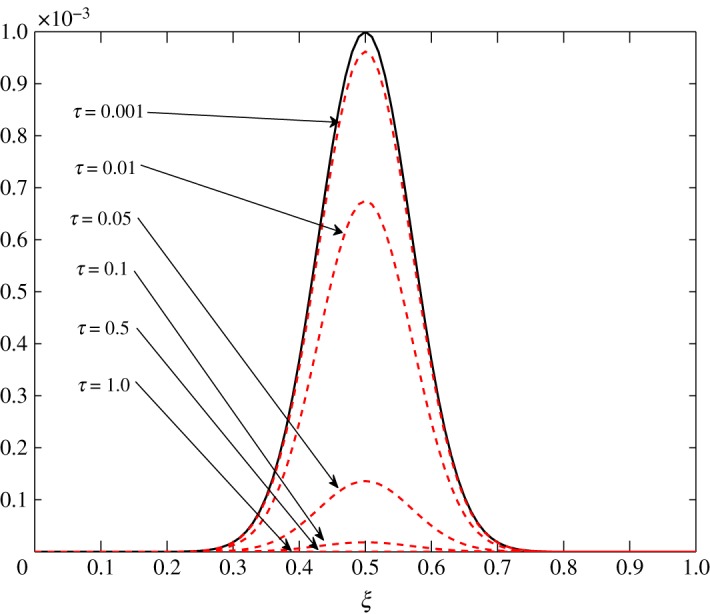
Relaxation of ${\hat{c}}_{1}$ in Case 1.

**Figure 5.**
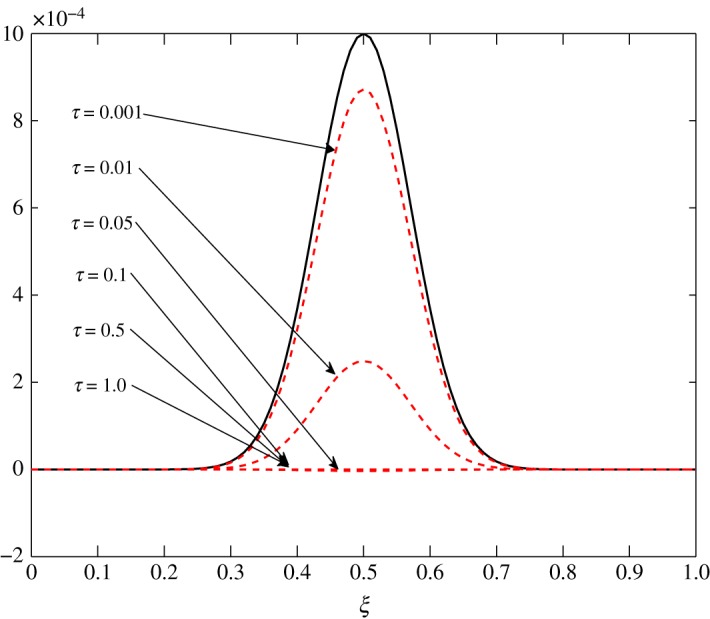
Relaxation of ${\hat{c}}_{2}$ in Case 1.

**Figure 6.**
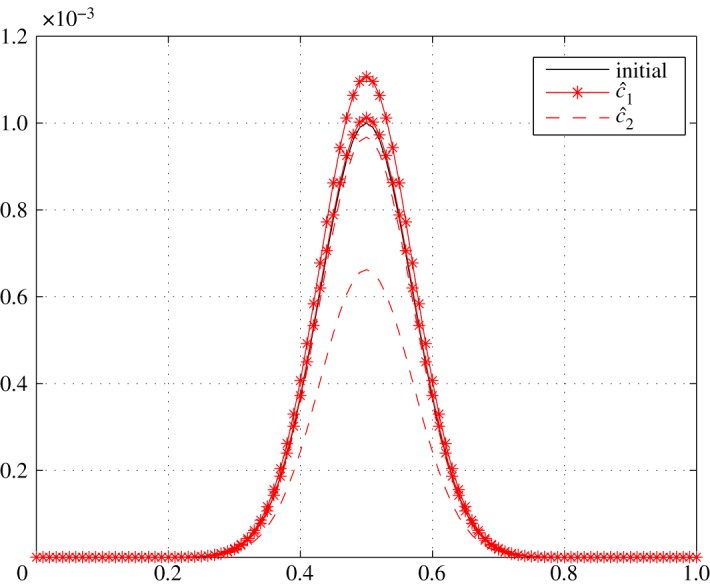
Relaxation effect of ${\hat{c}}_{1}$ and ${\hat{c}}_{2}$ on each other in Case 1.

From table 2, *r*_11_ shifts from a positive to a negative value somewhere in the range 0.4<*ξ*<0.5. An overshoot is also seen in the condition number, and the relaxation matrix is singular at that location as shown in figure 3. This transition can be considered as a site of exchange between air and vapour. At 0.4<*ξ*<0.5, *r*_11_ shifts from a positive to a negative value, that is, vapour concentration starts relaxing. The relaxation matrix becomes negative definite in this range and its determinant goes to zero. Figures 4 and 5 show the relaxation in the fluctuations of vapour and air in Case 1 at *τ*=0.001, 0.01, 0.05, 0.1, 0.5 and 1.0. A faster relaxation is observed with ${\hat{c}}_{2}$ when compared with ${\hat{c}}_{1}$, because *r*_22_<*r*_11_<0. The fluctuations in air reach zero faster. The contribution of ${\hat{c}}_{1}$ to the relaxation of ${\hat{c}}_{2}$ is negligible because |*r*_22_|≫|*r*_21_|. On the other hand, as *r*_12_>0 for *ξ*≥0.2, it reduces the relaxing capacity of ${\hat{c}}_{1}$. The cross effect of ${\hat{c}}_{1}$ and ${\hat{c}}_{2}$ on each other is illustrated in figure 6. A decreasing ${\hat{c}}_{2}$ results in an increasing ${\hat{c}}_{1}$ and that is due to the values of *r*_12_ and *r*_21_, as explained by (3.13) and (3.14).

**Table 2.** Analysing the signs of the elements in the coefficient matrices in Case 1.

process	influence of ${\hat{c}}_{1}$ on the evolution of ${\hat{c}}_{1}$	influence of ${\hat{c}}_{2}$ on the evolution of ${\hat{c}}_{1}$	influence of ${\hat{c}}_{1}$ on the evolution of ${\hat{c}}_{2}$	influence of ${\hat{c}}_{2}$ on the evolution of ${\hat{c}}_{2}$
relaxation	*r*_11_>0(*ξ*≤0.4) *r*_11_<0(*ξ*>0.4)	*r*_12_<0(*ξ*≤0.2) *r*_12_>0 (*ξ*>0.2)	*r*_21_<0	*r*_22_<0
transport	*t*_11_<0	*t*_12_<0	*t*_21_<0	*t*_22_<0
diffusion	*d*_11_>0	*d*_12_>0	*d*_21_>0	*d*_22_>0

#### 4.1.2. Transport process

**Figure 7.**
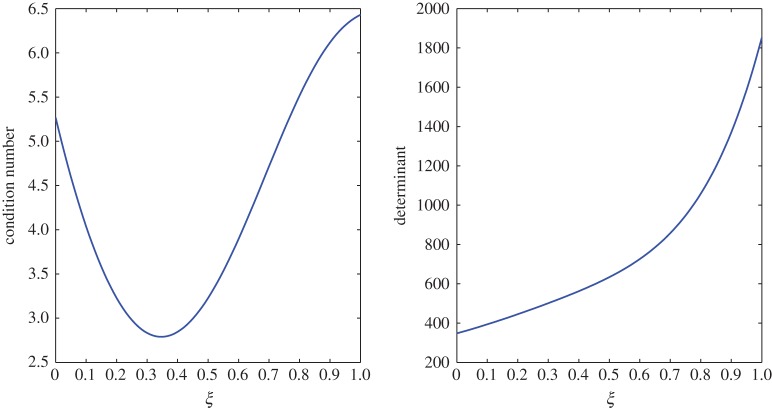
Condition number and determinant of the transport matrix in Case 1.

The large condition number of the transport matrix can pose a problem while inverting the matrix. However, the determinant suggests that the matrix is invertible at every point in the domain 0≤*ξ*≤1. The condition number and determinant of this transport matrix can be seen in Figure 7.

**Figure 8.**
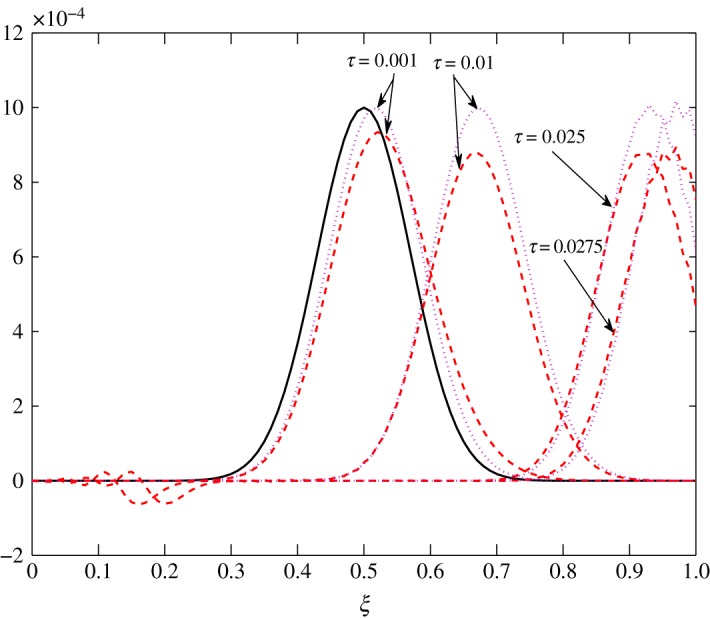
Advection of ${\hat{c}}_{1}$ in Case 1 at *τ*=0.001, 0.01, 0.025 and 0.0275.

**Figure 9.**
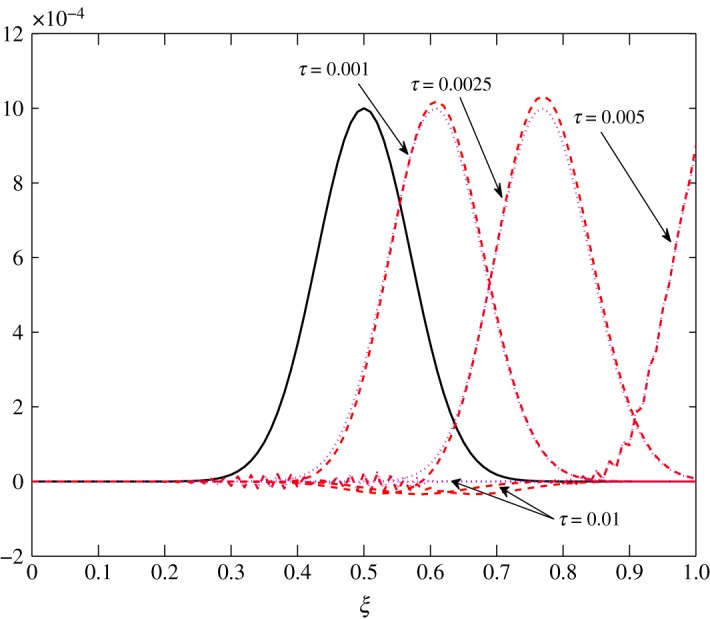
Advection of ${\hat{c}}_{2}$ in Case 1 at *τ*=0.001, 0.0025, 0.005 and 0.01.

*t*_12_ has a considerable effect on the advection of vapour. This is true because air motion provides extra resistance to vapour as explained by Huang *et al*. [10]. However, |*t*_21_|≪|*t*_22_| does not contribute much to the movement of air. From figures 8 and 9, the fluctuations in amplitude are due to the cross influence air and vapour have on each another. Pure advection of ${\hat{c}}_{1}$ and ${\hat{c}}_{2}$ is represented by the dotted curves. In such a case, the amplitude of the initial profile is preserved. A numerical method cannot be left untouched by numerical errors. The action of relaxation and diffusion tend to camouflage the dispersive nature of the numerical schemes, in the sense that they damp the oscillations produced. Numerical error, in terms of oscillations, can clearly be observed around 0.1<*ξ*<0.3 in figure 8 and 0.4<*ξ*<0.8 in figure 9.

#### 4.1.3. Diffusion process

**Figure 10.**
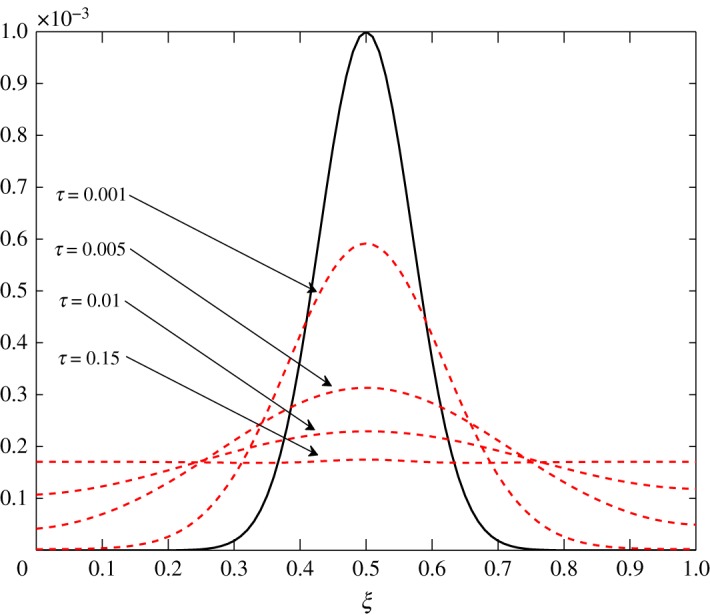
Diffusion of ${\hat{c}}_{1}$ in Case 1 at *τ*=0.001, 0.005, 0.01 and 0.15.

**Figure 11.**
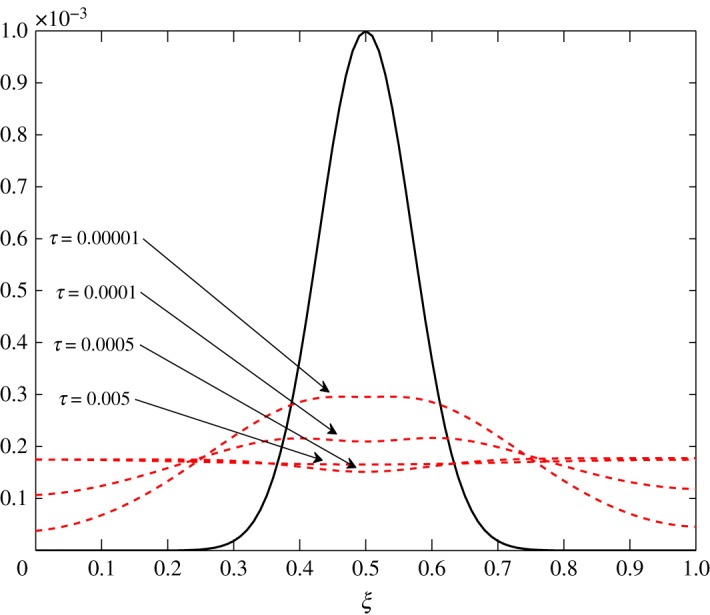
Diffusion of ${\hat{c}}_{2}$ in Case 1 at *τ*=0.00001, 0.0001, 0.0005 and 0.005.

As *d*_11_≈*d*_12_, ${\hat{c}}_{2}$ has an almost equal contribution in the diffusion of vapour. The bigger positive values of *d*_22_ mean that air damps at a faster rate compared to vapour, as seen in figures 10 and 11. Some over damping at 0.4<*ξ*<0.6 in figure 11 is a result of extra diffusion added by (3.25).

### 4.2. Case 2

**Figure 12.**
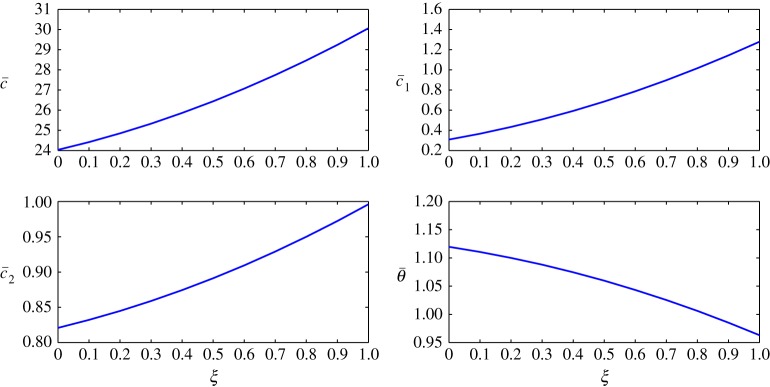
Profiles at steady state for Case 2.

A different scenario is taken, where the outer clothing layer is exposed to a higher level of humidity compared to the inner layer. As a result, an inverted situation is taken for the vapour concentration at steady state. The air concentration and temperature profiles of Case 1 are maintained. Figure 12 shows the profiles at steady state for this case. Such a situation can exist in reality during a cold rainy day. Table 3 summarizes the signs of the coefficient matrices for Case 2.

**Table 3.** Analysing the signs of the elements in the coefficient matrices in Case 2.

process	influence of ${\hat{c}}_{1}$ on the evolution of ${\hat{c}}_{1}$	influence of ${\hat{c}}_{2}$ on the evolution of ${\hat{c}}_{1}$	influence of ${\hat{c}}_{1}$ on the evolution of ${\hat{c}}_{2}$	influence of ${\hat{c}}_{2}$ on the evolution of ${\hat{c}}_{2}$
relaxation	*r*_11_<0	*r*_12_<0 (*ξ*<0.1, 0.7<*ξ*≤1.0) *r*_12_>0 (0.1≤*ξ*≤0.7)	*r*_21_<0	*r*_22_<0
transport	*t*_11_<0 (*ξ*≤0.2) *t*_11_>0 (*ξ*>0.2)	*t*_12_>0	*t*_21_>0	*t*_22_>0
diffusion	*d*_11_>0	*d*_12_>0	*d*_21_>0	*d*_22_>0

#### 4.2.1. Relaxation process

**Figure 13.**
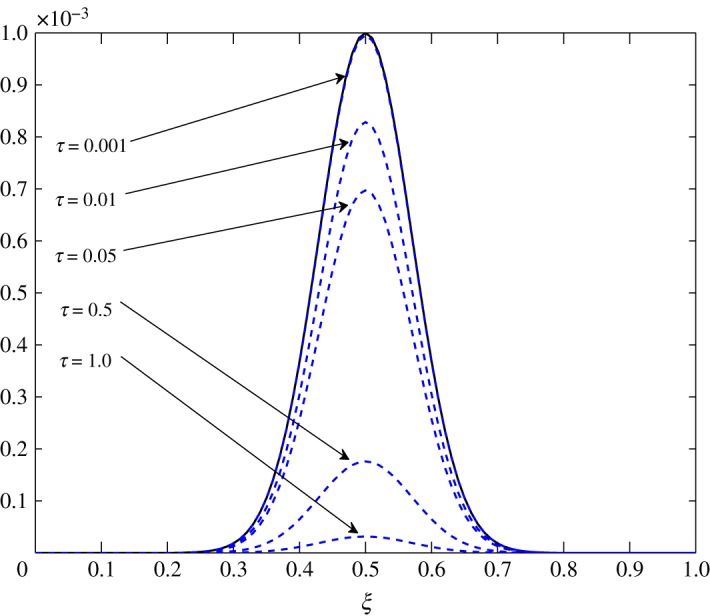
Relaxation of ${\hat{c}}_{1}$ in Case 2 at *τ*=0.001, 0.01, 0.05, 0.5 and 1.0.

**Figure 14.**
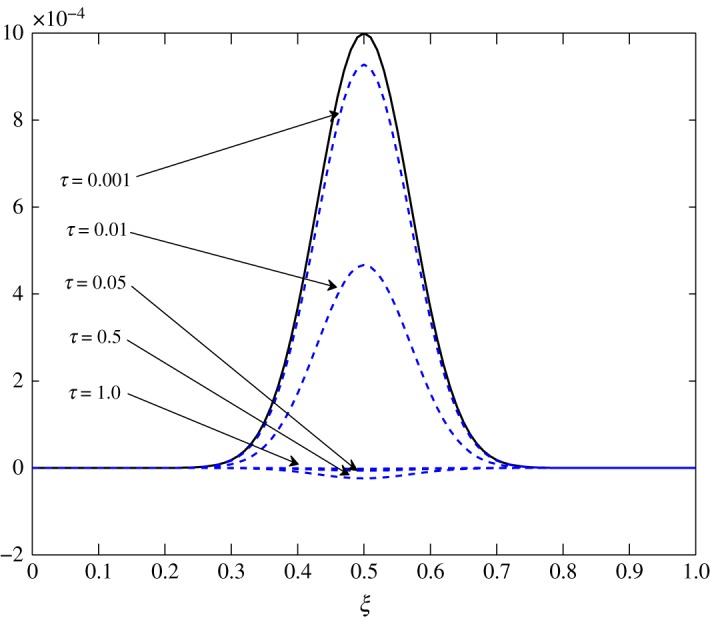
Relaxation of ${\hat{c}}_{2}$ in Case 2 at *τ*=0.001, 0.01, 0.05, 0.5 and 1.0.

Referring to (3.11) and (3.12), *r*_11_<0 and *r*_22_<0 mean that both air and vapour concentrations undergo natural relaxation. Nevertheless, *r*_22_<*r*_11_<0 results in air concentration relaxing faster than that of vapour. Over relaxation may also occur due to the large relaxation coefficients of ${\hat{c}}_{2}$. The positive values of *r*_12_ in the range 0.1≤*ξ*≤0.7 reduce the relaxation of ${\hat{c}}_{1}$. The above is observed in figures 13 and 14. Clearly, ${\hat{c}}_{1}$ does not relax to zero in this case as seen in figure 13. This is in fact logical because there will always exist vapour in the batting. The high level of humidity outside and the production of sweat by the body maintains a certain concentration of vapour in the batting, and hence fluctuations will always exist.

**Figure 15.**
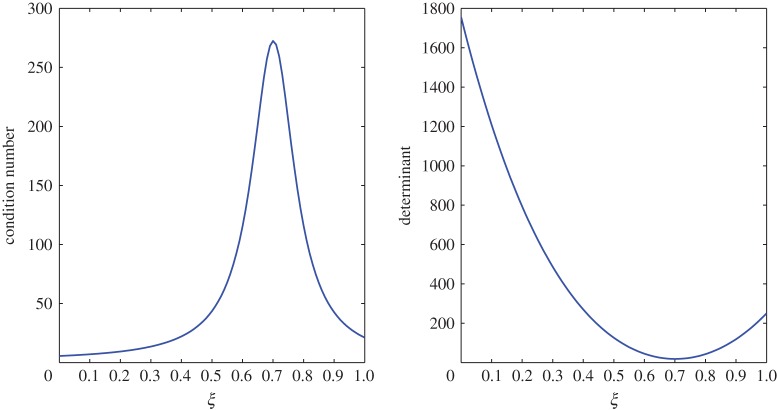
Condition number and determinant of the relaxation matrix in Case 2.

In this case, the relaxation matrix is invertible at every point in the batting. However, at around *ξ*=0.7 where the transition in *r*_12_ takes place, a peak is observed in the condition number and the determinant reaches a minimum point as shown in figure 15.

#### 4.2.2. Transport process

**Figure 16.**
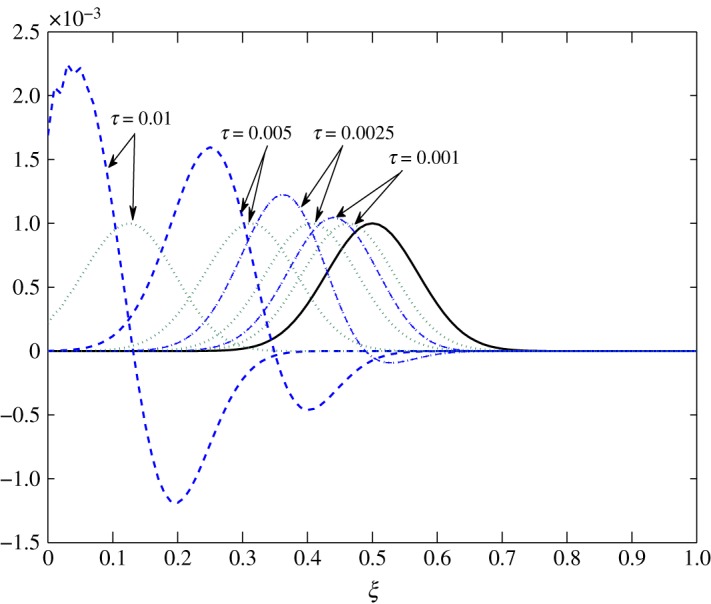
Transport of ${\hat{c}}_{1}$ in Case 2 at *τ*=0.001, 0.0025, 0.005 and 0.01.

**Figure 17.**
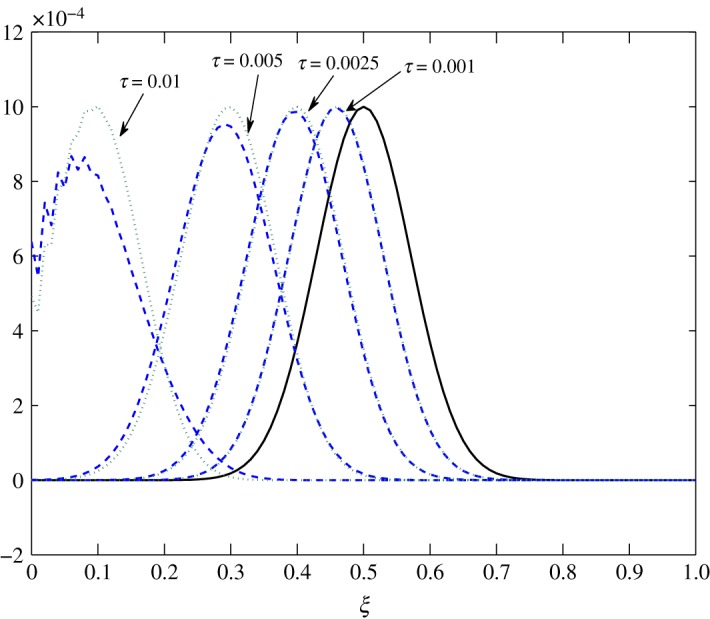
Transport of ${\hat{c}}_{2}$ in Case 2 at *τ*=0.001, 0.0025, 0.005 and 0.01.

The transport process in Case 2 is very distinct from that of Case 1. Almost all elements are positive, which implies advection in the opposite direction. Figures 16 and 17 show the evolution of ${\hat{c}}_{1}$ and ${\hat{c}}_{2}$ within the batting. Here, the fluctuations ${\hat{c}}_{1}$ and ${\hat{c}}_{2}$ travel to the l.h.s., that is, towards the body. Table 3 points out the negative values of *t*_11_ at *ξ*≤0.2. Thus, ${\hat{c}}_{1}$ has a tendency to move away from the body. However, as *t*_12_>0, ${\hat{c}}_{2}$ has a dominant influence on the advection of ${\hat{c}}_{1}$ which prevents this movement. As the elements are almost of the same order, air and vapour fluctuations move at almost the same speed.

**Figure 18.**
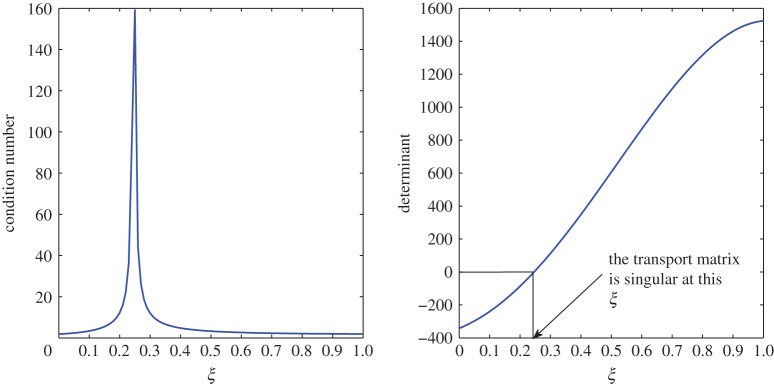
Condition number and determinant of the transport matrix in Case 2.

In this case, the transport process hints at an exchange site between air and vapour, which is shown in figure 18. In the range 0.2<*ξ*<0.3, the determinant becomes zero, and this is where *t*_11_ shifts from negative to positive. In the previous case, this phenomenon was noted in the relaxation process.

#### 4.2.3. Diffusion process

**Figure 19.**
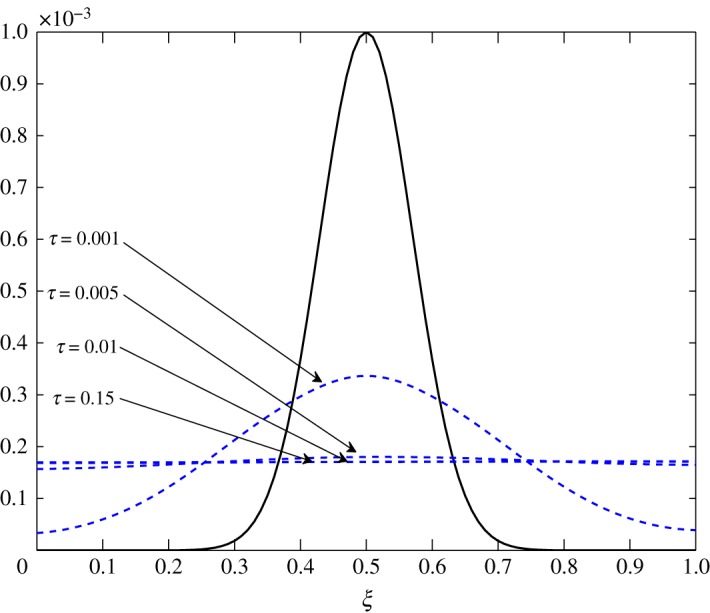
Diffusion of ${\hat{c}}_{1}$ in Case 2 at *τ*=0.001, 0.005, 0.01 and 0.15.

**Figure 20.**
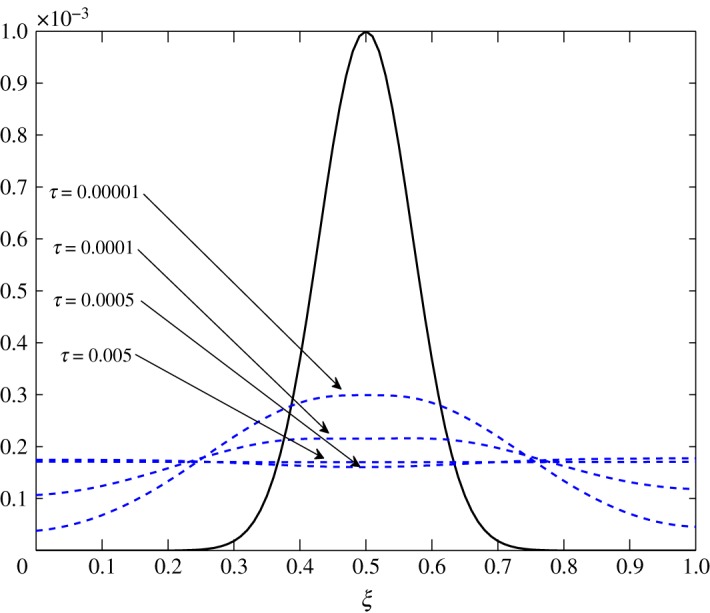
Diffusion of ${\hat{c}}_{2}$ in Case 2 at *τ*=0.00001, 0.0001, 0.0005 and 0.005.

The diffusion process is the least affected process by the change made at steady state. The trend of the elements is similar to those in Case 1, *d*_21_ and *d*_22_ being unaffected by this change. The only difference observed in table 3 is in the values corresponding to the evolution of ${\hat{c}}_{1}$. All positive elements suggest damping of the fluctuations as seen in figures 19 and 20. In this case, ${\hat{c}}_{1}$ damps more as it approaches the outer environment because of the increasing values of *d*_11_ and *d*_12_ as ξ→1.0. Again, ${\hat{c}}_{2}$ damps quicker than ${\hat{c}}_{1}$.

This case is also dissipative in nature. The diffusion and relaxation offer enough damping to avoid the growth of the fluctuations over time.

### 4.3. Case 3

**Figure 21.**
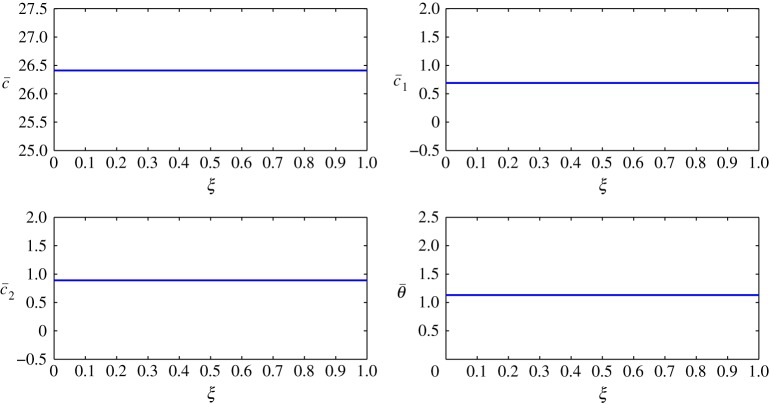
Profiles at steady state for Case 3.

The final situation considered is when the vapour and air concentrations, and temperature are constant at steady state. That is, the internal and external environments are exposed to the same conditions. The profiles of such a situation is given in figure 21. All processes are constant throughout the batting. The coefficient matrices are given in table 4.

**Table 4.** Analysing the values of the elements in the coefficient matrices in Case 3.

process	influence of ${\hat{c}}_{1}$ on the evolution of ${\hat{c}}_{1}$	influence of ${\hat{c}}_{2}$ on the evolution of ${\hat{c}}_{1}$	influence of ${\hat{c}}_{1}$ on the evolution of ${\hat{c}}_{2}$	influence of ${\hat{c}}_{2}$ on the evolution of ${\hat{c}}_{2}$
relaxation	*r*_11_<0	*r*_12_=0	*r*_21_=0	*r*_22_=0
transport	*t*_11_=0	*t*_12_=0	*t*_21_=0	*t*_22_=0
diffusion	*d*_11_>0	*d*_12_>0	*d*_21_>0	*d*_22_>0

#### 4.3.1. Relaxation process

**Figure 22.**
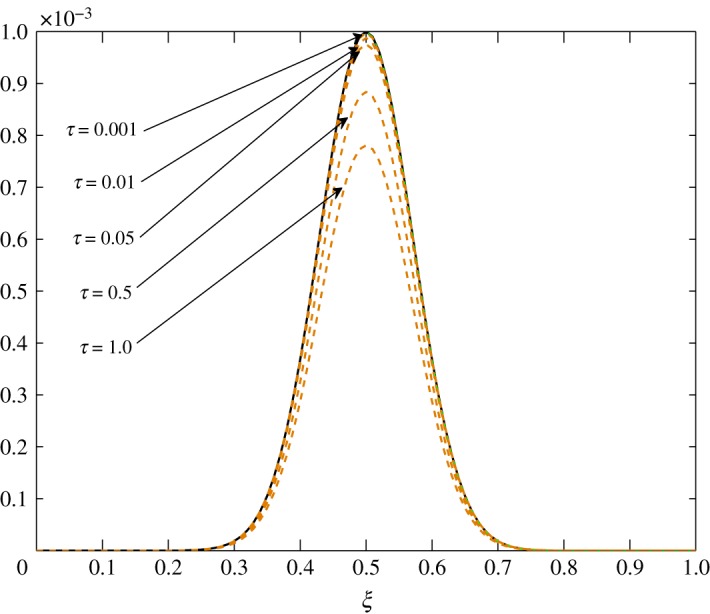
Relaxation of ${\hat{c}}_{1}$ in Case 3 at *τ*=0.001, 0.01, 0.05, 0.5 and 1.0.

The only non-zero element is *r*_11_. This is caused by the last term in the first element of the relaxation coefficient, in (3.1). The value of $-(\beta_{G}/\epsilon )A\sqrt{\bar{\theta }}$ gives ${\hat{c}}_{1}$ a slight relaxing power. In spite of this, very little relaxation is observed in the fluctuations of vapour, as seen in figure 22. Evidently, no change happens to ${\hat{c}}_{2}$.

#### 4.3.2. Transport process

In this case, the fluctuations do not travel to either side of the batting. The zero coefficient elements suggest that ${\hat{c}}_{1}$ and ${\hat{c}}_{2}$ remain stationary.

#### 4.3.3. Diffusion process

**Figure 23.**
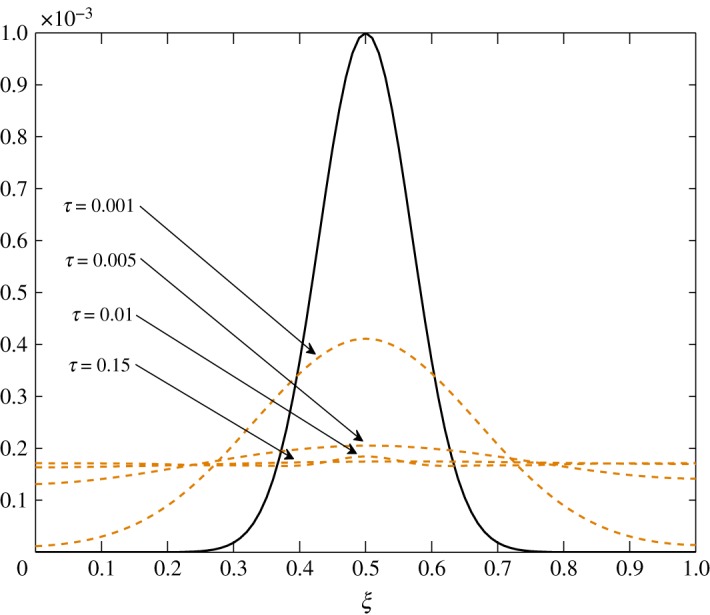
Diffusion of ${\hat{c}}_{1}$ in Case 3 at *τ*=0.001, 0.005, 0.01 and 0.15.

**Figure 24.**
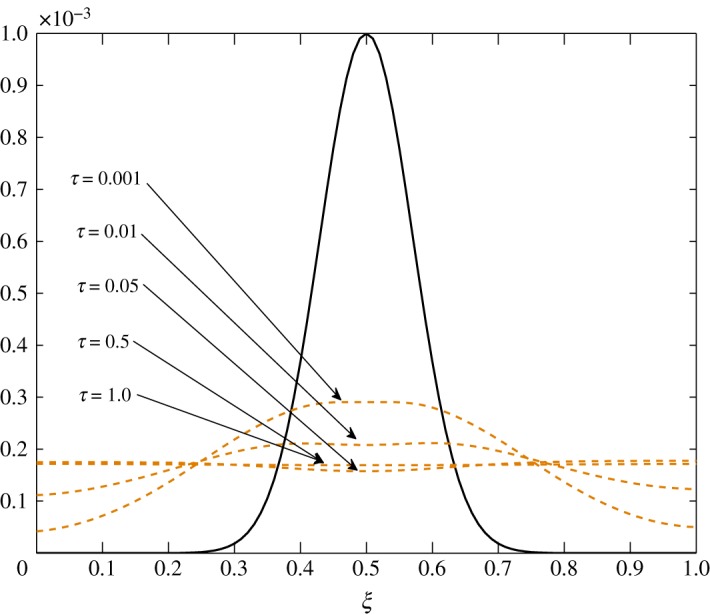
Diffusion of ${\hat{c}}_{2}$ in Case 3 at *τ*=0.001, 0.01, 0.05, 0.5 and 1.0.

The only visible process in this situation is diffusion. A uniform damping takes place. ${\hat{c}}_{2}$ has an equal contribution in the diffusion of $\hat{c}{{}_{1}}_{\tau }$ because *d*_11_≈*d*_12_. As observed in the earlier cases, air damps more rapidly than vapour. This is seen in figures 23 and 24.

Next, the combined behaviour of all three processes in each of the three different cases is investigated.

### 4.4. Relaxation–transport–diffusion process

The three physical processes in some way compensate for the numerical error produced by the finite difference method, as no oscillation is noticed. The numerical results with respect to to vapour concentration are obtained at *τ*=0.001, 0.005, 0.01, 0.05 and 1.0. The evolution of air concentration is approximated at *τ*=0.00005, 0.0001, 0.001, 0.01 and 1.0.

**Figure 25.**
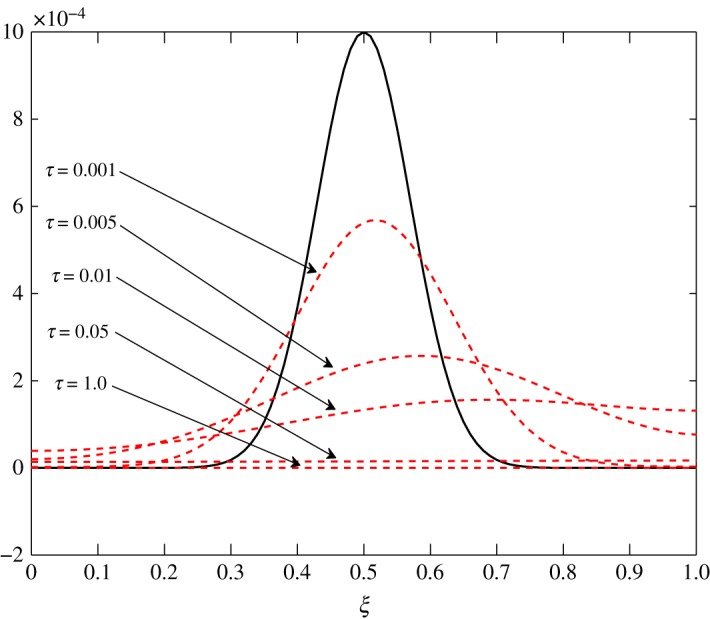
Relaxation–transport–diffusion phenomena of ${\hat{c}}_{1}$ in Case 1.

**Figure 26.**
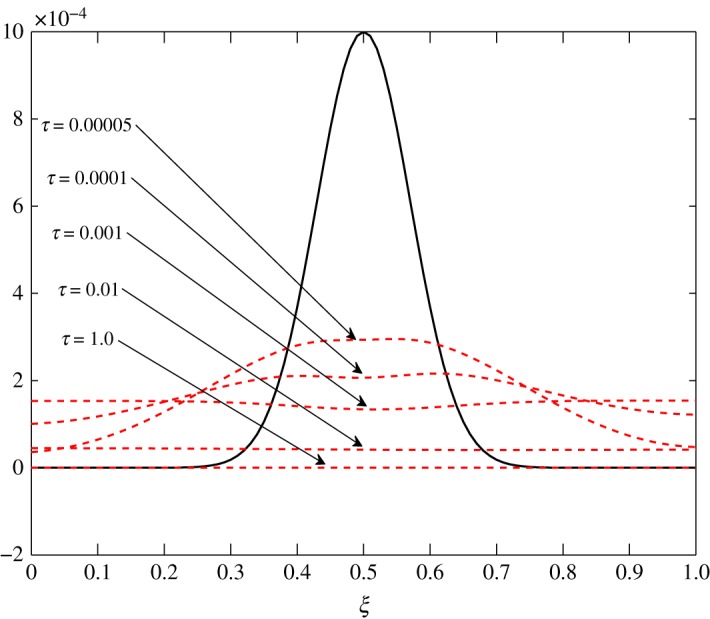
Relaxation–transport–diffusion phenomena of ${\hat{c}}_{2}$ in Case 1.

**Figure 27.**
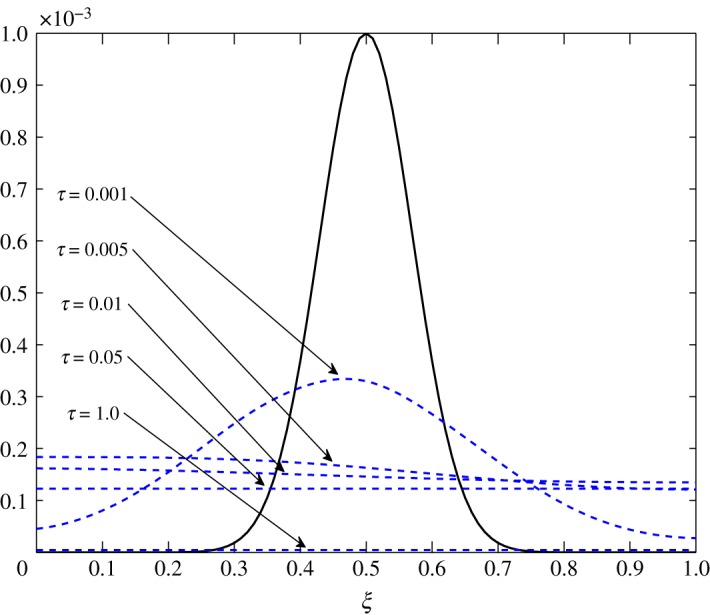
Relaxation–transport–diffusion phenomena of ${\hat{c}}_{1}$ in Case 2.

**Figure 28.**
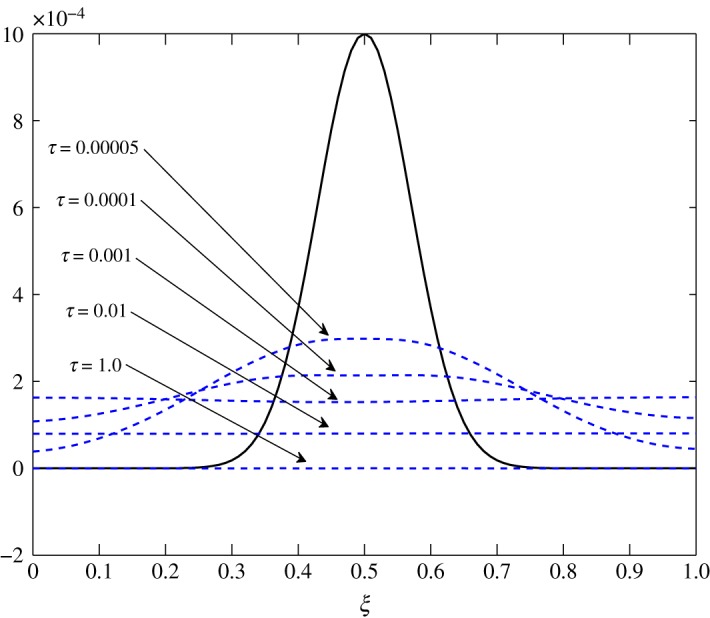
Relaxation–transport–diffusion phenomena of ${\hat{c}}_{2}$ in Case 2.

**Figure 29.**
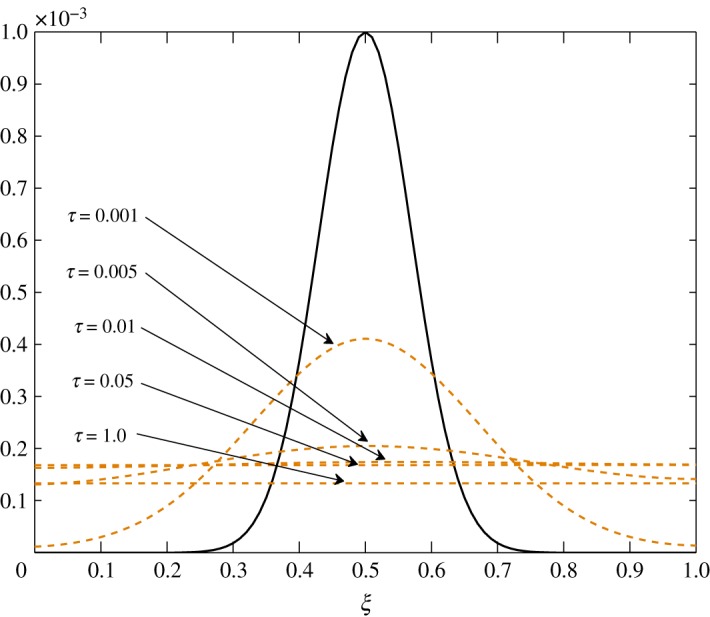
Relaxation–transport–diffusion phenomena of ${\hat{c}}_{1}$ in Case 3.

**Figure 30.**
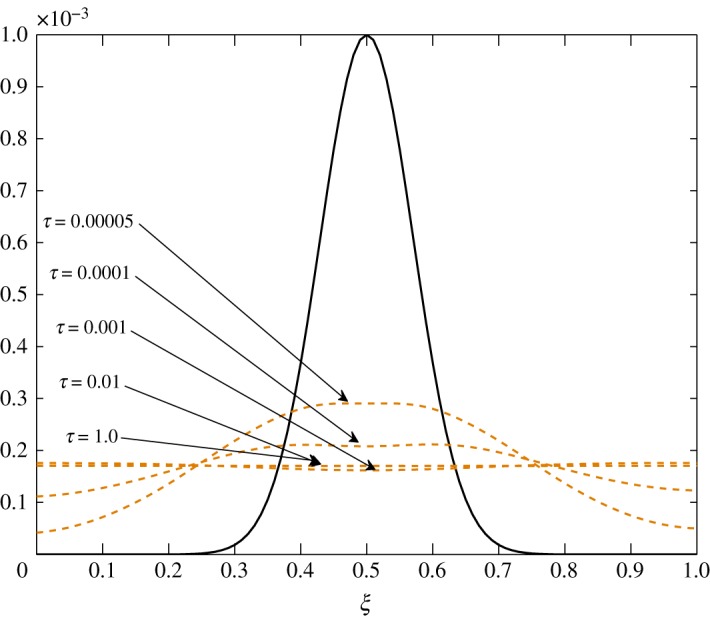
Relaxation–transport–diffusion phenomena of ${\hat{c}}_{2}$ in Case 3.

From figures 25 and 26, advection to the r.h.s. is seen. Similarly, figures 27 and 28 show movement to the l.h.s. In figures 29 and 30, a symmetric dissipation is observed because of the absence of the advection process in Case 3. Case 1 shows the fluctuations tending to zero over time. However, fluctuations in vapour will always exist in Case 2. In Case 3, the fluctuations in vapour and air concentration do not tend to zero.

In figures 31–39, the initial fluctuations are made to vary sinusoidally as follows:

$${\hat{c}}_{1}(\xi ,0)=0.05\sin(50\pi \xi )\;\text{and}\;{\hat{c}}_{2}(\xi ,0)=0.005\sin(50\pi \xi ).$$

**Figure 31.**
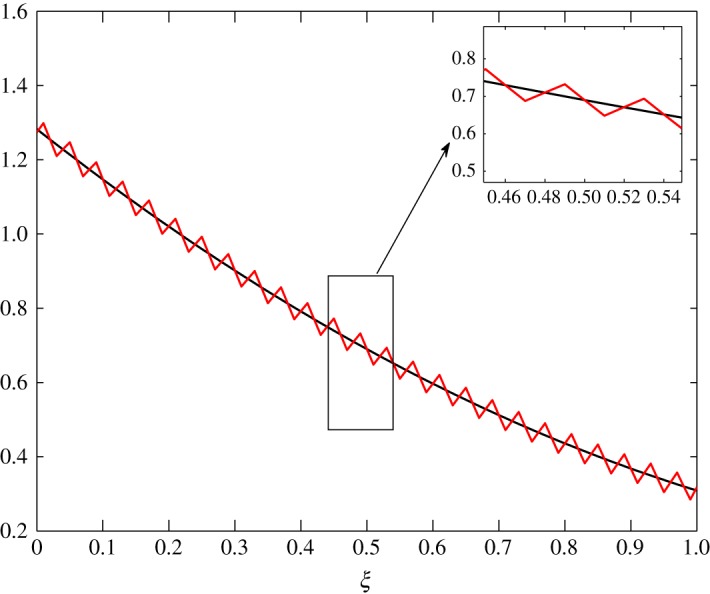
Evolution of ${\hat{c}}_{1}$ at *τ*=0.01 in Case 1.

**Figure 32.**
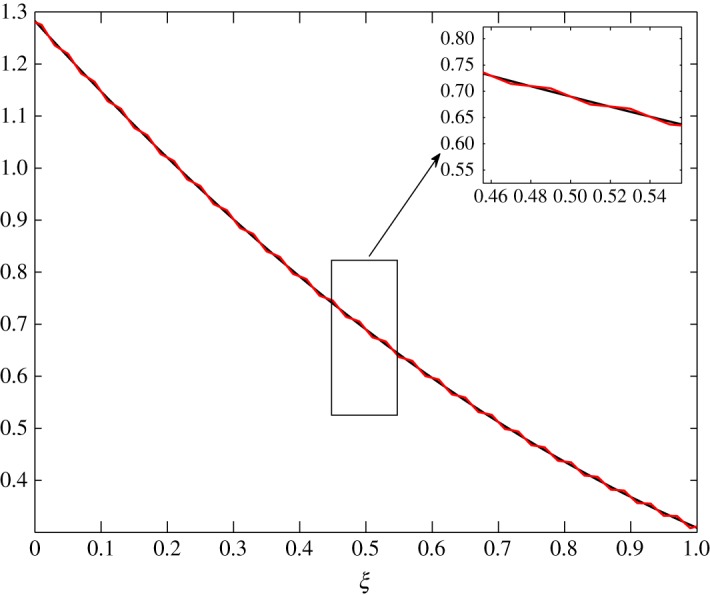
Evolution of ${\hat{c}}_{1}$ at *τ*=0.05 in Case 1.

**Figure 33.**
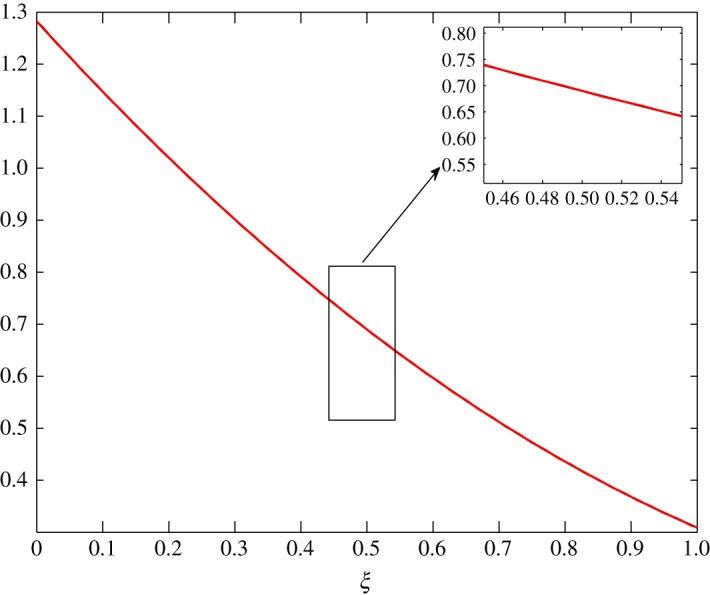
Evolution of ${\hat{c}}_{1}$ at *τ*=0.1 in Case 1.

**Figure 34.**
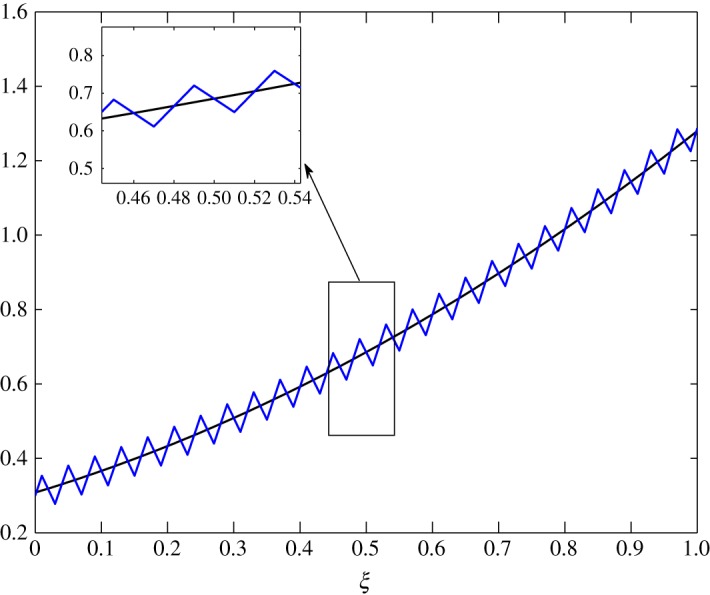
Evolution of ${\hat{c}}_{1}$ at *τ*=0.01 in Case 2.

**Figure 35.**
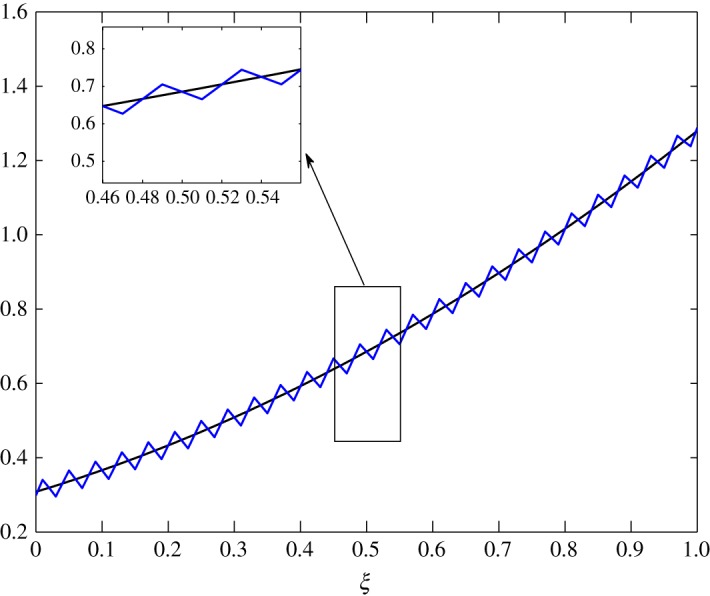
Evolution of ${\hat{c}}_{1}$ at *τ*=0.05 in Case 2.

**Figure 36.**
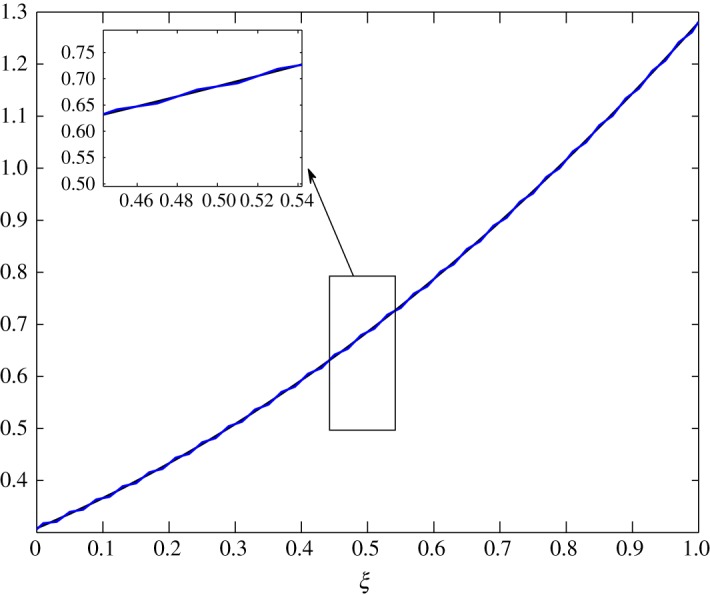
Evolution of ${\hat{c}}_{1}$ at *τ*=0.25 in Case 2.

**Figure 37.**
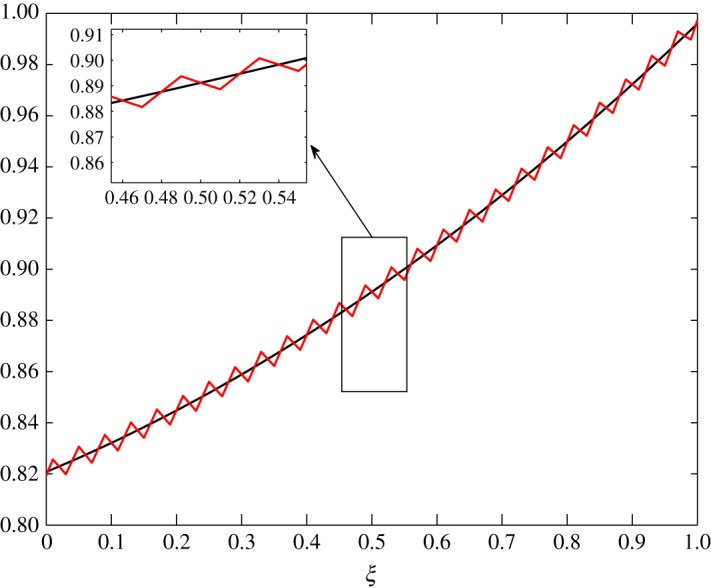
Evolution of ${\hat{c}}_{2}$ at *τ*=0.0001 in Case 1.

**Figure 38.**
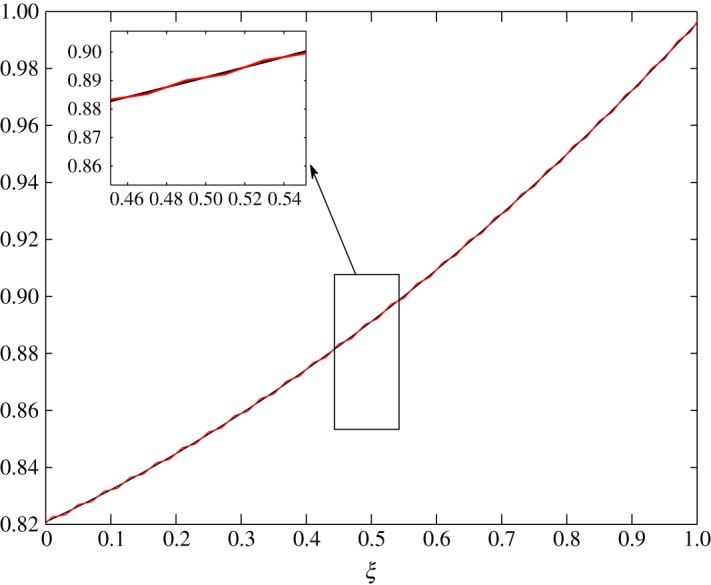
Evolution of ${\hat{c}}_{2}$ at *τ*=0.0005 in Case 1.

**Figure 39.**
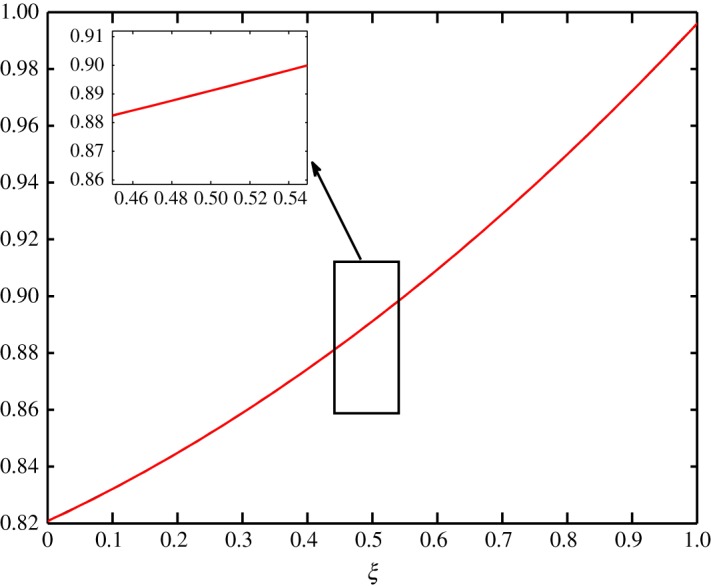
Evolution of ${\hat{c}}_{2}$ at *τ*=0.001 in Case 1.

Figures 31–33 show the evolution of vapour concentration in Case 1. As desired, the fluctuations die out over time. The initial configuration is thus recovered.

Nevertheless, $\lim_{\tau \to 0}{\hat{c}}_{1}\neq 0$ in Case 2. Figures 34–36 show the fluctuations damping with time, but a small amount of fluctuation will always exist in the batting.

The evolution of ${\hat{c}}_{2}$ is similar in Case 1 and 2. Air concentration diffuses and relaxes very quickly while being transported out of the polyester batting.

As observed in Cases 1–3, the steady state has an important influence on the relaxation, transport and diffusion processes. The slightest change in profile of any one parameter at steady state influences the evolution of ${\hat{c}}_{1}$ and ${\hat{c}}_{2}$ significantly.

The relaxation, transport and diffusion processes provide useful insight on the choice of fabric with respect to the environmental conditions. In this study, the three-layered clothing assembly is ideal for the situation in Case 1. The 10-layer polyester batting damps fluctuations in both air and vapour concentrations completely, hence giving the wearer a uniform feel. The clothing assembly also constantly eliminates wetness in the fabric, as seen in figures 31–33, providing better comfort. In Case 2, the relaxation and transport phenomena do not permit complete evacuation of sweat. In places of high humidity, a fabric with higher vapour resistance at the cover layers should be chosen. An appropriate clothing assembly should ensure a negative transport matrix in (3.1). A three-layered clothing assembly is not appropriate in Case 3. With a constant temperature of 303K between the body and environment, a thinner and hygroscopic garment will be preferable.

## 5. Conclusion

In this work, the model given in Huang *et al*. [10] is considered. The evolution of fluctuations in vapour and air under the influence of heat in a 10-layer polyester batting, at steady state, is the site of investigation. The processes of relaxation, transport and diffusion of the fluctuations of vapour and air concentrations at steady state are investigated. A complex nonlinear system of Petrovskii parabolic PDE resulted from this formulation. To analyse the resulting PDE, the profiles at steady state were linearized. The importance of these profiles was depicted in three cases where it was clearly noticed that a small change made at steady state influenced the three phenomena significantly. It is shown that the system has a positive and unique solution, and would always remain bounded due to its dissipative nature. The semi-implicit Crank–Nicolson was chosen to solve the system of PDEs numerically. Numerical investigation supported the arguments made for the three distinct cases, highlighting the importance of the steady-state values in the evolution of the fluctuation of air and vapour concentration, respectively. Ideally, a clothing assembly should filter out the fluctuations and advect the vapour away from the skin. The fabric properties, which are incorporated in the values at steady state, determine its effectiveness when exposed to various conditions. It is seen that the clothing assembly with negative relaxation, negative transport and positive diffusion matrices is more capable of eliminating the fluctuations.

The fluctuations that are inherent to both the human and environmental conditions were the main focus of this study, hence the one-dimensional setting used. However, in reality the fabric in a clothing assembly may not be distributed uniformly and hence the heat and moisture transfer may not be isotropic in the clothing. As a result, a three-dimensional model is more realistic. This limitation will be addressed in future studies. In addition, the model is currently being extended in order to accommodate actual fluctuations in the ambient temperature.

## Data accessibility

All the experimental parameters used in this paper have been cited from Fan *et al.* [9] and Huang *et al.* [10]. The data available in these two cited articles are sufficient for a reader to replicate our work.
